# Gut microbiota associated with appetite suppression in high-temperature and high-humidity environments

**DOI:** 10.1016/j.ebiom.2023.104918

**Published:** 2023-12-16

**Authors:** Yalan Wu, Xiangrong Feng, Mengjun Li, Zongren Hu, Yuhua Zheng, Song Chen, Huanhuan Luo

**Affiliations:** a Research Centre of Basic Integrative Medicine, School of Basic Medical Sciences, Guangzhou University of Chinese Medicine, Guangzhou, Guangzhou, China; b Department of Rehabilitation and Healthcare, Hunan University of Medicine, Hunan, China; c Science and Technology Innovation Center, Guangzhou University of Chinese Medicine, Guangzhou, China; d State Key Laboratory of Traditional Chinese Medicine Syndrome, Guangzhou University of Chinese Medicine, Guangzhou, China

**Keywords:** Gut microbiota, Appetite suppression, High-temperature and high-humidity environments, Mendelian randomization analyses

## Abstract

**Background:**

Food is crucial for maintaining vital human and animal activities. Disorders in appetite control can lead to various metabolic disturbances. Alterations in the gut microbial composition can affect appetite and energy metabolism. While alterations in the gut microbiota have been observed in high-temperature and high-humidity (HTH) environments, the relationship between the gut microbiota during HTH and appetite remains unclear.

**Methods:**

We utilised an artificial climate box to mimic HTH environments, and established a faecal bacteria transplantation (FMT) mouse model. Mendelian randomisation (MR) analysis was used to further confirm the causal relationship between gut microbiota and appetite or appetite-related hormones.

**Findings:**

We found that, in the eighth week of exposure to HTH environments, mice showed a decrease in food intake and body weight, and there were significant changes in the intestinal microbiota compared to the control group. After FMT, we observed similar changes in food intake, body weight, and gut bacteria. Appetite-related hormones, including ghrelin, glucagon-like peptide-1, and insulin, were reduced in DH (mice exposed to HTH conditions) and DHF (FMT from mice exposed to HTH environments for 8 weeks), while the level of peptide YY initially increased and then decreased in DH and increased after FMT. Moreover, MR analysis further confirmed that these changes in the intestinal microbiota could affect appetite or appetite-related hormones.

**Interpretation:**

Together, our data suggest that the gut microbiota is closely associated with appetite suppression in HTH. These findings provide novel insights into the effects of HTH on appetite.

**Funding:**

This work was supported by the 10.13039/501100001809National Natural Science Foundation of China and 10.13039/501100010618Guangzhou University of Chinese Medicine.

**Research in context**

**Evidence before this study**

Recently, the impact of the environment on human health has been attracting increasing attention. Long-term exposure to high humidity and heat results in high morbidity and mortality rates. These conditions can also cause dehydration, heat stress, and loss of appetite. Altered gut microbiota has been observed in the HTH environments and may play a pivotal role in regulating appetite. The mechanisms underlying intestinal bacteria and appetite suppression in HTH environments remain an active research topic. Moreover, Mendelian randomisation analysis provides a favourable method for exploring the causal relationship between intestinal bacteria and appetite.

**Added value of this study**

Our study utilised an artificial climate box to mimic the HTH environments and explored the relationship between the gut microbiome and appetite in HTH environments. We identified a key role for the gut microbiota in appetite inhibition in the HTH environments. Long-term exposure to HTH altered the gut microbial composition in mice, decreased food intake and body weight, and affected the expression of appetite-related hormones. MR analysis was also used to further indicate a causal relationship between these changes in the intestinal microbiota and appetite or appetite-related hormones.

**Implications of all the available evidence**

Available evidence suggests that the gut microbiota exerts an influential role in appetite inhibition in the HTH environments, and the altered expression of appetite-related hormones may represent a key factor in their impact on appetite. The findings of this study contribute to the growing body of evidence supporting the notion that hazards to human health are caused by long-term exposure to HTH environments. In the future, it may be possible to reduce the weight of people with obesity through FMT or probiotic treatment. Despite these promising findings, further research is required to determine their safety and efficacy. In summary, temperature and humidity were investigated to generate experimental data on the effects of climatic factors on the microbiota and host health.

## Introduction

With global warming, the frequency and intensity of heat waves are gradually increasing.^1^ Early research primarily focused on the effects of high temperatures on health, but relative humidity is also crucial when defining heat waves because of its relationship with the human body heat exchange.^2^, ^3^, ^4^ Recent studies have suggested that the risks associated with heatwaves under humid conditions may surpass those associated with temperature, intensifying the apparent temperature peaks and the strength of heatwaves.^5^ Long-term exposure to high-temperature and high-humidity (HTH) environments can cause various health concerns. Owing to relative humidity rise, the efficiency of sweat cooling reduced, increasing the body’s heat burden and resulting in high morbidity and mortality rates.^3^^,^^5^ These conditions can also cause dehydration, heat stress, and loss of appetite. Wang et al. showed significant elevation in the levels of leptin and peptide YY (PYY) in HTH groups, suggesting that these conditions might disrupt the neural system and appetite regulation.^6^ Despite the acceleration of metabolism by HTH, high temperatures result in an increase in core body temperature, causing loss of appetite and reduced energy intake.^7^, ^8^, ^9^, ^10^

Appetite is regulated by the central nervous system and responds to short-term signals from gastrointestinal hormones and long-term signals from the adipose tissue, which are related to energy storage and environmental cues.^11^ The gastrointestinal tract and its microbiota play key roles in this process. Physiological control of appetite is mediated by circulating orexigenic and anorexigenic hormones (e.g., leptin, insulin, and ghrelin) produced by peripheral organs, including the gut, adipose tissue, and pancreas.^12^ The impact of the gut microbiome on host energy metabolism and related diseases has drawn significant attention,^13^^,^^14^ including changes in the gut microbial composition in obesity,^15^, ^16^, ^17^ anorexia nervosa,^18^, ^19^, ^20^ and severe forms of acute malnutrition, such as marasmus.^21^ Gut microbes not only utilise host energy but also provide metabolic products, such as short-chain fatty acids and amino acids, for host energy.^22^ Research has shown that gut microbes affect many appetite pathways,^23^, ^24^, ^25^ including regulating food digestion and absorption, influencing satiety with metabolic products, and interacting with the immune system.^26^ The composition of the gut microbiome is closely related to the feeding behaviours of humans and animals.^22^^,^^27^ Altering the gut microbial composition through dietary interventions or probiotics can affect appetite and energy metabolism of the host.^28^ Alterations in the gut microbiota have been observed in HTH environments,^29^^,^^30^ However, the relationship between the gut microbiome and appetite has not yet been clarified.

Mendelian randomization is a statistical method based on whole genome sequencing data that can effectively reduce bias and reveal causal relationships.^31^ The genetic variation associated with a certain risk factor is used as a instrumental variable to infer the causal effect of the exposure factor on the outcome of interest.^32^ This is similar to a randomized controlled trial where one group of individuals carries specific genetic variations (exposure group), while the other group does not carry these variations (non exposure group). Compared to traditional observational studies, this natural randomization helps alleviate confounding issues. Due to the difference between the exposed and non-exposed groups, it is more likely to have a causal relationship with the outcome of interest.^33^

Here, we performed 16S rRNA gut microbiome sequencing studies in mice exposed to HTH and normal environments, as well as faecal bacterial transplantation (FMT). We verified changes in appetite-related hormones in the serum after exposure to HTH environments. Furthermore, we revealed associations between the gut microbiome and appetite using Mendelian randomisation (MR). These results highlight the role of gut microbiota in regulating appetite and provide novel insights into the impact of HTH environments on appetite.

## Methods

### Animal experimental design

Male BALB/c mice (6-week-old, male) were obtained from and maintained at the Laboratory Animal Research Center of Guangzhou University of Chinese Medicine (GZUCM, Guangzhou, China). All mice were housed in sterile autoclaved cages with irradiated food and acidified autoclaved water. Animals were housed in sterile cages in a room maintained at 25 ± 1 °C, with an average humidity of 60–65% and a 12-/12-h light/dark cycle and provided access to water and rodent chow ad libitum. All experiments were approved by the Animal Experimental Ethical Committee of the Traditional Chinese Medicine Hospital of Zhongshan (No. 2018007). All procedures were performed according to the recommendations of the National Institutes of Health Guide for the Care and Use of Laboratory Animals National Research Council Guide for the Care and Use of Laboratory Animals.

**Fig. 1:**
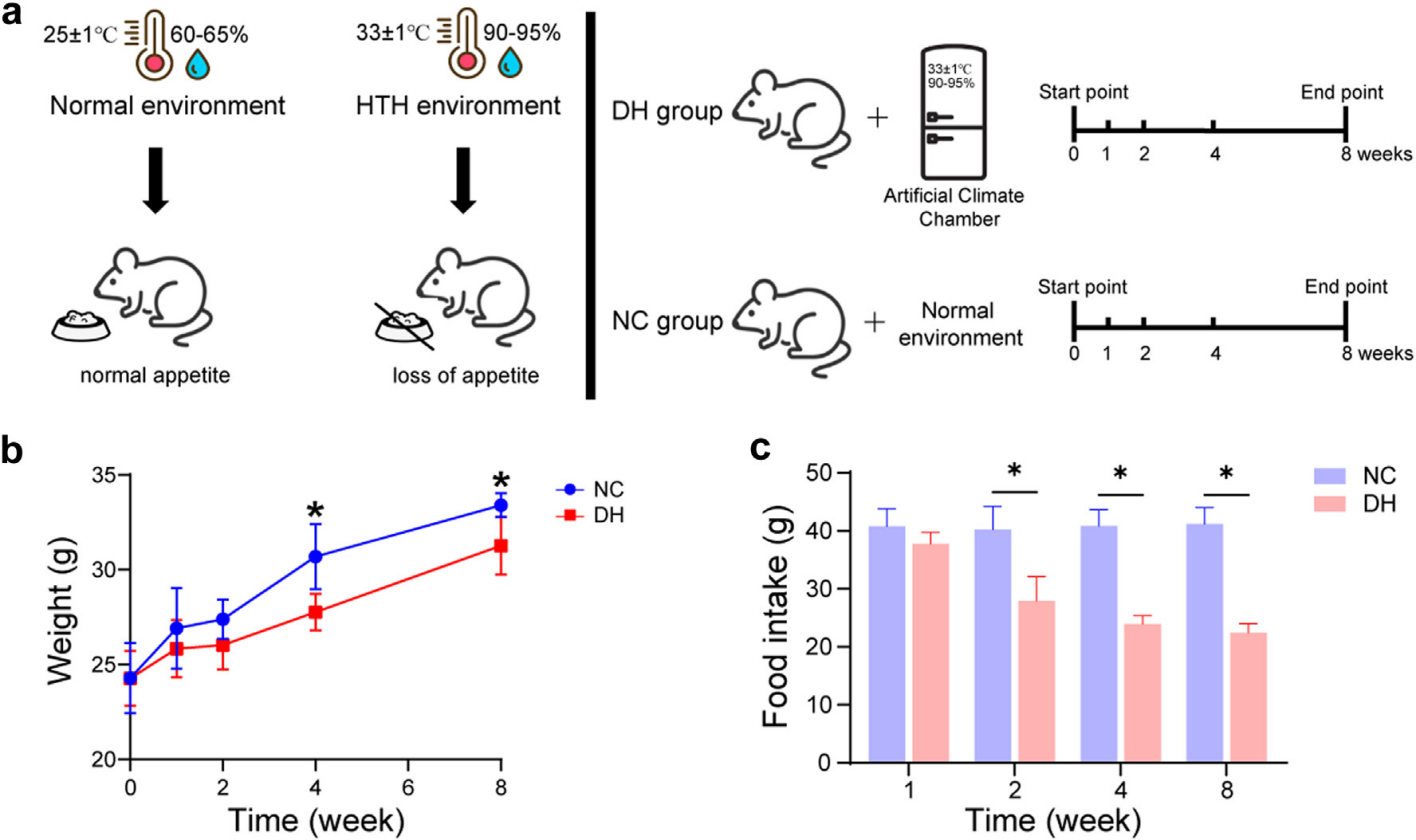
**The alteration of body weight and food intake in mice exposed to HTH environments.(a)** The animal experimental protocol. **(b)** Body weight in the DH and NC groups at weeks 1, 2, 4 and 8. **(c)** Food intake in the DH and NC groups at weeks 1, 2, 4 and 8. Average daily food intake of each cage of mice for a week. DH, mice exposed to HTH environments; NC, mice exposed to normal environments (n = 6 for all groups).

The experimental protocol is shown in Fig. 1a. Mice were randomly assigned to an experimental group (DH) and a control group (NC). The control group did not receive any treatment. The experimental group was exposed to HTH conditions (temperature 33 ± 1 °C, relative humidity 90–95%) via an artificial climate box (model: RXZ-158A, Ningbo Jiangnan Instrument Factory, China).^34^

The animal experimental protocol for the FMT is shown in Fig. 3a. Mice were randomly divided into two groups: treatment group (DHF, FMT from mice exposed to the HTH environments for 8 weeks) and control group (NF, FMT from mice exposed to normal environments). Both the DHF and NF groups were housed in sterile cages in a room maintained at 25 ± 1 °C with an average humidity of 60–65%. Faecal suspensions were prepared as previously described.^35^ Briefly, fresh faecal pellets (200 mg) from mice exposed to normal environments or HTH environments for 8 weeks were resuspended in 5 mL saline solution, vigorously shaken for 3 min, and allowed to settle by gravity for 2 min. FMT administration was performed by gavage with 200 μL of the supernatant from the faecal sample for a period of 2 weeks (a total of six times) in the DHF and NF groups.

**Fig. 2:**
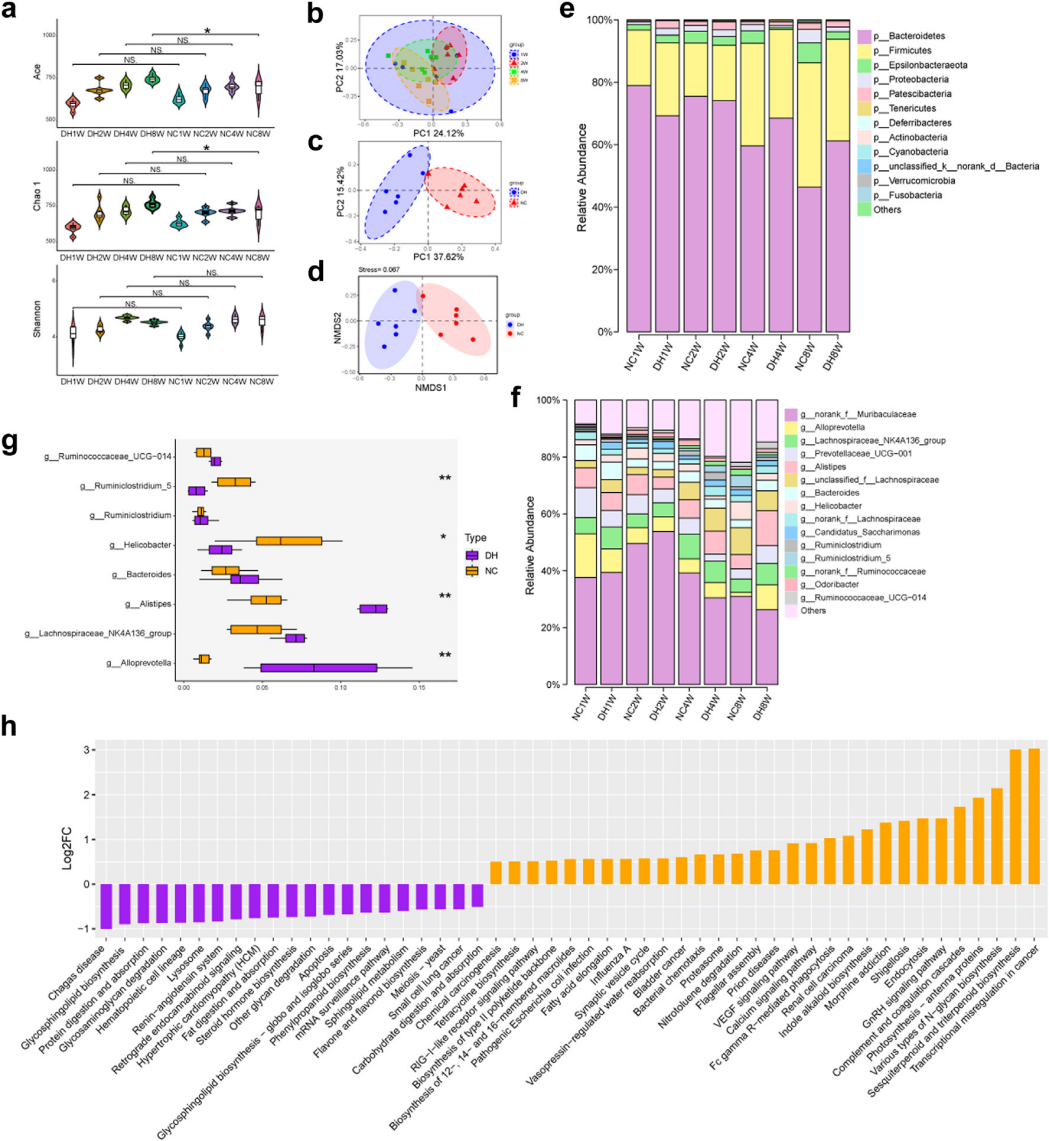
**The alteration of gut microbiome in mice exposed to HTH environments.(a)** Species diversity differences between the DH and NC groups were estimated by the Ace, Shannon, and Chao1 indices. ∗*P* < 0.05; NS., not significant (n = 6 for all groups). **(b and c)** PCoA plot base of the relative abundance of OTUs by Unweighted UniFrac distance (n = 6 for all groups). **(b)** The DH group at weeks 1, 2, 4 and 8; **(c)** the DH and NC groups at week 8. **(d)** NMDS plot of the relative abundance of OTUs between the DH and NC groups at week 8 (n = 6 for all groups). **(e and f)** Component proportion of bacteria from fecal content 16S rRNA sequencing data between the DH and NC groups at week 8 (Wilcoxon rank-sum test) (n = 6 for all groups). **(e)** The phylum level (top 12); **(f)** the genus level (top 15). **(g)** Comparison of relative abundance of bacterial taxa at the genus level between the DH and NC groups at week 8. The Wilcoxon rank sum test was used to determine the significance between groups. ∗*P* < 0.05, ∗∗*P* < 0.01 vs. the NC group. **(h)** Pathway enrichment and statistical significance of the 51 metabolites that were present in the DH and NC groups at week 8. Statistical significance was evaluated using *P*-values adjusted for multiple comparisons with the False Discovery Rate (FDR) method. In all figures, ∗*P* < 0.05, ∗∗*P* < 0.01, and ∗∗∗*P* < 0.001 denote FDR-adjusted levels of significance.

**Fig. 3:**
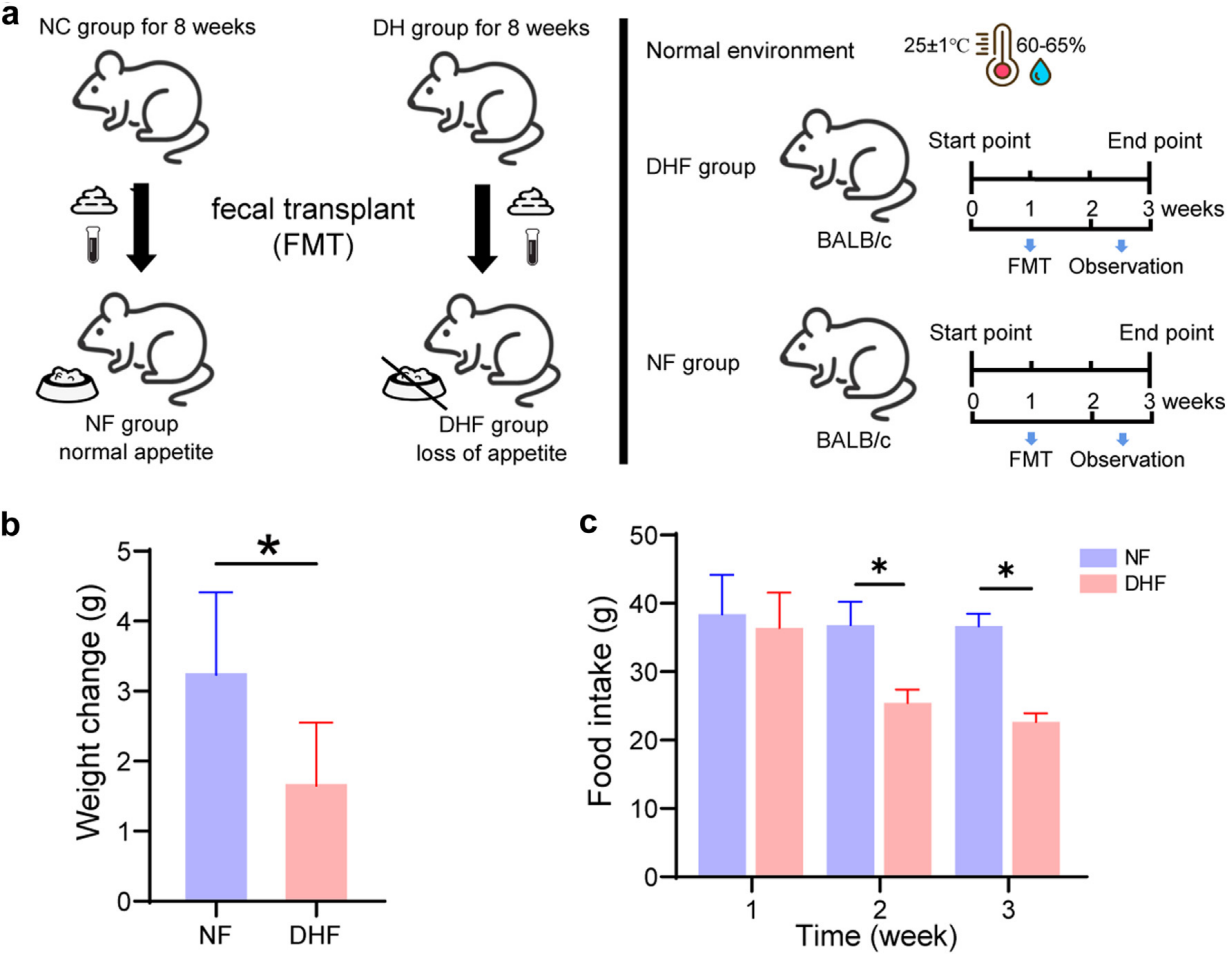
**The alteration of body weight and food intake in mice after FMT treatment.(a)** The animal experimental protocol for FMT. **(b)** Body weight in the DHF and NF groups. **(c)** Food intake in the DHF and NF groups. Average daily food intake of each cage of mice for a week. DHF, FMT from mice exposed to HTH environments for 8 weeks (n = 10); NF, FMT from mice exposed to normal environments (n = 13).

### Faecal DNA extraction and 16S rRNA sequencing and data analysis

Faecal samples were collected and stored at −80 °C until bacterial DNA isolation and extraction were performed according to previously reported methods.^36^ Primers were designed based on the conserved regions of the target genes, and sequencing adaptors were added at their ends.^37^^,^^38^ Microbial genes were amplified using the forward primer 515F 5′-GTGCCAGCMGCCGCGGTAA-3′ and the reverse primer 806R 5′-GGACTACHVGGGTWTCTAAT-3′. To produce amplicon libraries, a 30 μL reactions with 15 μL of Phusion® High-Fidelity PCR Master Mix (New England Biolabs); 0.2 μM of forward and reverse primers was used, containing 10 ng of each genomic DNA sample, 1.25 U of Taq DNA polymerase, 5 μL of 10 × Ex Taq buffer, 10 mM of dNTPs (all reagents were sourced from Takara Biotechnology Co., Ltd., Dalian, China) and 40 pmol of primer mixture. PCR conditions were as follows: initial denaturation at 98 °C for 1 min; 30 cycles of denaturation at 98 °C for 10 s, annealing at 50 °C for 30 s, and extension at 72 °C for 30 s; followed by a final extension at 72 °C for 5 min. PCR products were purified with GeneJET Gel Extraction Kit (Thermo Scientific). The concentration of the amplicon libraries was estimated using the a 2100 Bioanalyzer system (Agilent Technologies GmbH, Waldbronn, Germany), and equal amounts of amplicons from each sample were pooled together.

Raw data were processed using an internal pipeline developed based on Mothurv 1.31.2.^39^ Primers were removed, and low-quality sequences were truncated (sequences with an average quality score <20 over a 30-bp window), pooling all high-quality reads from a single sample that was longer than 250-bp. Subsequently, operational taxonomic units (OTUs) were clustered using USEARCH (v7.0.1090) with 97% identity.^40^ The relative abundances of the OTUs were then calculated. OTUs with relative abundances of <0.001% were discarded and the distribution of OTUs in each sample was analysed. Both weighted and unweighted UniFrac distance analyses were performed based on OTU abundance and phylogenetic trees. For α-diversity analysis, the vegan package (2.6.4) was used to compute diversity indices. PCoA and NMDS were employed for β-diversity analysis. In addition, PICRUSt was used to perform functional classification of KEGG. Alpha and beta diversity analyses were performed using the R software package (version 4.2.3).

### ELISA

ELISA kits for mouse ghrelin (E-EL-M0551c; Elabscience), insulin (E-EL-M1382c, Elabscience), GLP-1 (E-EL-M3012; Elabscience), and PYY (E-EL-M2375c; Elabscience) were purchased from BGsciences Biotechnology Co., Ltd. (Guangzhou, China). ELISA was performed using serum samples. Blood samples were obtained from heart and allowed to clot for 30 min at 37 °C. After centrifugation at 2000*g* for 15 min, the serum was collected from the blood samples. Serum was used for ELISA. The ELISA was performed according to the manufacturer’s instructions. The standard curves were plotted for each experiment.

### MR

MR analyses were performed in R using the TwoSampleMR software package (v.0.5.7). In this study, we focused on the causal relationships between 131 gut microbiota features and changes in appetite (specifically manifested as “recent poor appetite or overeating”) as well as certain appetite hormones (leptin, PYY, GLP-1, and insulin). The inverse variance-weighted method, MR-Egger, and weighted median MR sensitivity analyses were used to identify the microbial taxa related to appetite. Additionally, we used the simple mode and weighted mode to estimate the causal effect of the gut microbiota on appetite and appetite-related hormones. To confirm the reliability of our MR causal estimates, we also performed multiple sensitivity analyses, including examination of level pleiotropy (MR-Egger intercept, *P* > 0.05), heterogeneity (Cochran’s Q test, *P* > 0.05), and leave-one-out analysis.^41^ MR employs key statistical indicators to assess the relationships. These include odds ratio (OR), which are used to gauge the degree of association between genetic variants and specific traits or diseases. An OR value greater than 1 indicates a positive correlation, an OR value less than 1 indicates a negative correlation, and an OR value equal to 1 indicates no correlation. The farther the OR value is from 1, the stronger the correlation. Beta values indicate the strength and direction of the relationships in continuous traits. The standard error (SE) quantifies the estimation uncertainty. 95% confidence interval of odds ratio measures the credibility range, where the inclusion of 1 signifies a non-significant association, and its absence implies significance. Two-sample MR (TS-MR) was initially conducted to identify and prioritise the gut microbiota significantly associated with appetite. Ultimately, using a multistep MR design, a causal pathway from gut microbiome taxa to appetite, mediated by appetite-regulating hormones, was established.

### Data sources and research participants

Summary statistics for the exposure and outcome phenotypes are derived from previous GWAS studies, as listed in Supplementary Table S14. The study data for the gut bacterial taxa included 113 species at the genus level, which were used as exposures. The comprehensive GWAS summary data primarily originated from the largest meta-analysis of gut microbiome compositions to date conducted by the MiBioGen consortium (Kurilshikov et al., 2021). This study encompassed 18,340 individuals from 24 cohorts, targeting the V4, V1–V2, and V3–V4 variable regions of the 16S rRNA gene to delineate microbial composition and employed direct taxonomic classification methods. Localisation analyses were conducted on microbiota quantitative trait loci to ascertain host genetic variation and locate genetic loci associated with bacterial abundance in the gut microbiome (Sanna et al., 2019). It recorded 211 gut microbial taxa and 122,110 associated single nucleotide polymorphisms (SNPs) (accessed from https://mibiogen.gcc.rug.nl/menu/main/home on 1 August 2023) (Kurilshikov et al., 2021). GWAS summary statistics for “poor appetite or overeating” are sourced from UK Biobank (Pan-UKB team, 2020) for a meta-analysis related to the question, “Over the last 2 weeks, how often have you been bothered by any of the following problems? [Depressive symptoms] Poor appetite or overeating”, involving 157,298 South Asian participants (https://gwas.mrcieu.ac.uk/datasets/ukb-e-20511_CSA/). Data for PYY is derived from a GWAS meta-analysis (https://www.ebi.ac.uk/ols/ontologies/efo/terms?iri=http%3A%2F%2Fwww.ebi.ac.uk%2Fefo%2FEFO_0007937&lang=en&viewMode=All&siblings=false) titled “blood protein measurement” from the Experimental Factor Ontology with an unknown sample size, consisting of 501,428 SNPs, all of which are of European ethnicity. GLP-1 data stems from the GWAS Catalogue’s meta-analysis (https://www.ebi.ac.uk/gwas/studies/GCST005353) titled “GLP-1-stimulated insulin secretion”, a study of 126 individuals on a whole-genome genotyping chip.^42^ Gudmundsdottir et al. conducted an inaugural GWAS on GLP-1 stimulated insulin secretion in non-diabetic individuals (n = 126) from the Netherlands Twin Register, correlating network-prioritised SNP polygenic risk scores with glucose-stimulated insulin secretion phenotypes in up to 5318 individuals in the MAGIC cohort (*P* < 0.05). Data for Leptin originates from IEU OpenGWAS (https://gwas.mrcieu.ac.uk/datasets/ieu-a-1003/), where Kilpelainen et al. conducted a GWAS on circulating leptin levels in 32,161 individuals, and a follow-up on gene loci achieving *P* < 10^−6^ in another 19,979 individuals, all of European descent.^43^ Insulin data were derived from the GWAS atalogue (https://www.ebi.ac.uk/gwas/studies/GCST006675), representing the largest GWAS on first-phase insulin secretion, conducted by Wood AR, measured via an intravenous glucose tolerance test, and involved up to 5567 non-diabetic individuals from 10 studies of Hispanic or Latin American descent.^44^

### Genetic tool variable selection

We employed genetic variants of microbial taxon units with a *P*-value threshold (<1 × 10^−5^) from GWAS testing and an effect allele frequency of >0.01. The instrument variable inclusion threshold of *P* < 1 × 10^−5^ was determined by maximising the genetic variance explained by genetic predictors, as used in previous gut microbiome MR studies.^45^ Independent SNPs were selected as instrumental variables based on r^2^ < 0.001 in the 1000 Genomes Europe dataset. When there were no shared SNPs between exposure and outcome, surrogates from the 1000 Genomes Europe data were added (r^2^ > 0.8). Only results based on at least three shared SNPs were retained. The Wald ratio was used for MR causality testing, and the inverse variance weighted (IVW) method was used for meta-analysis of the Wald ratio.^46^ Causality was estimated using other methods: the weighted-pattern method,^47^ which provides an alternative method for IVW, and the MR-Egger method,^41^ which estimates the degree of horizontal pleiotropy in the data. We utilised a genome-wide significance *P*-value threshold (<5 × 10^−8^) during the selection of genetic instrument variables for appetite hormones and appetite.^48^, ^49^, ^50^ Subsequently, all these genetic variations were aggregated within ±10,000-kb distances with a linkage disequilibrium threshold of r^2^ < 0.001 using the 1000 Genomes European reference panel.^50^ For palindromic SNPs, the forward strand allele was ascertained using the allele frequency information. If an SNP was absent in the outcome GWAS summary data, proxies with r^2^ ≥ 0.8 as substitutes were employed. Finally, the instrumental variable strength was estimated using the F-statistic, and the amount of variance explained by the instrumental variable for each exposure was calculated using the TwoSampleMR software package (get_r_from_lor function) for binary traits and phenotypic variation, as defined by Shim et al. for quantitative traits. The F statistic was then calculated as $\frac{r^{2}\times \left(N-1-k\right)}{(1-r^{2})\times k}$, where r^2^ 'is the explained variance, N is the sample size, and k is the number of instrumental variables.^51^ The conventional threshold of F-statistic >10 was retained.^52^ After performing MR testing, we duplicate GWAS traits were excluded because the same phenotype was usually observed in multiple GWAS. To remove duplicates, samples for each trait in all the tested GWAS were retained. After excluding duplicates and tests performed with weak instrumentation (F statistic <10), BH correction for multiple testing was applied to the results obtained from the IVW MR test and subsequently strict filtering procedures were used to avoid false positives.

### Statistical analysis

All statistical analyses were performed using R software package (version 4.2.3). For microbiome classification, the RandomForest package (version 4.7.1.1) was used for random forest modelling. Microbial taxon data from the DH and NC groups were used as the training set to build the random forest model. After the model was successfully established, the sample data from the NF and DHF groups were tested to compare the degree of fit of the test set in the random forest model. To evaluate the classification efficacy of the model, we plotted a receiver operating characteristic (ROC) curve and calculated the area under the curve (AUC). To validate the robustness of the model, we conducted 10-fold cross-validation. In addition, we assessed the importance of each feature in the model and filtered them based on the MeanDecrease Gini metric. For differences between continuous variables, we applied the Student’s t-test (for comparisons between two groups), Wilcoxon signed-rank test (for paired samples), and Kruskal–Wallis test (for comparisons among three or more groups). We also conducted an exhaustive Spearman correlation analysis to determine the relationship between the bacterial species and appetite hormones. To control for Type I errors due to multiple testing, we implemented the Benjamini and Hochberg method for FDR correction. FDR values below 0.05 were considered statistically significant.

### Role of funders

Funders had no input on study design, data collection, data analyses, interpretation, or writing of report.

## Results

### The alteration of body weight and food intake in mice exposed to HTH environments

We simulated an HTH living environment (relative humidity 90–95%, temperature 33 ± 1 °C) using an artificial climate control box and conducted a humid heat modelling for 8 weeks. In the present investigation, the body weight of the mice showed an increasing trend with increasing modelling time. However, mice exposed to HTH conditions (DH) had slower body weight gain and lower food intake than mice surviving under normal conditions (NC), and there was a significant difference between weeks 4 and 8 (Fig. 1b and c, Supplementary Table S6).

### Altered gut microbiota diversity in mice exposed to HTH environments

To evaluate the differences in bacterial diversity between the two groups, sequences were aligned to estimate alpha and beta diversity; there were statistically significant differences in the Ace and Chao1 indices, whereas the Shannon index was not significantly different between the DH and NC groups (Fig. 2a). Differences in principal component analysis between the two groups were observed using PCoA of Bray–Curtis distance (Fig. 2b and c), which was significant at week 8, according to PERMANOVA analysis. In addition, differences in beta diversity were observed via NMDS analysis of Bray–Curtis, revealing a separation between the two groups (Fig. 2d, Supplementary Figure S1d–f). A significant difference was observed at week 8 between the unweighted PCoA, compared to that at weeks 1, 2, and 4 (Fig. 2c; Supplementary Figure S1a–c). These results suggest that the diversity of the gut microbiota is strongly influenced by HTH.

### Alterations in the composition of faecal microflora in mice exposed to HTH environments

The relative proportions of the dominant taxa at the phylum and genus levels were assessed via microbial taxa assignment in both groups (Fig. 2e and f). We observed considerable variability in the gut microbiota across samples from the DH and NC groups at different exposure times. The most significant difference in the microbial community was observed at the week 8, compared to that in the NC group. The microbiomes of both DH and NC mice were dominated by Firmicutes and Bacteroidetes, which are typical gut microbiome structures in mice (Fig. 2e). At week 8, Bacteroidetes was the most prominent gut bacterial community, accounting for an average of 46.4% and 61.2% of the sequences in the NC and DH mice, respectively. Firmicutes represented the second most dominant gut bacterial community, accounting for an average of 39.9% and 32.7% of the sequences in the NC and DH mice, respectively. Moreover, we found that the proportion of Firmicutes showed a gradual increase as the duration of exposure to damp heat increased, but there was no significant difference compared with the NC group. In contrast, there was a decreasing trend in Bacteroidetes, and there was a significant difference compared with the NC group (Fig. 2e). At the family level, the relative abundance of *Rikenellaceae* and *Prevotellaceae* significantly increased in the DH group at the week 8 according to the Wilcoxon rank-sum test (Supplementary Figure S1g and k). At the genus level, there was a significant difference between the DH and NC groups at week 8 (Fig. 2f and g, Supplementary Figure S1h–j). In the present study, enrichment of *Alloprevotella*, *Alistipes* and *Lachnospiraceae_NK4A136_group* as well as depletion of *Ruminiclostridium_5*, was observed in DH mice (Fig. 2g, Supplementary Table S10) (*P* < 0.05, *P* < 0.01). *g__Ruminococcaceae_UCG-014* was also increased in the DH group, and there was no significant difference compared to the NC group. To further understand the impact of high-humidity and high-heat environments on intestinal microbial function, we used PICRUSt to predict the functional composition of all samples. Using Desq2 for differential analysis, 51 functional pathways with significant differences were identified between the two groups and |log2FC| > 0.5. Multiple KEGG (level 3) functional categories were affected in the DH group. Particularly in the DH group at week 8, significant differences were observed in pathways, such as the pentose phase pathway, pentose and glucose conversion, fructose and mannose metabolism, and galactose metabolism (Fig. 2h, Supplementary Table S9). Notably, pathways related to glucose metabolism were significantly disrupted in the DH group. These results suggest that HTH may have a profound effect on sugar metabolism, which may also be a key factor in decreased appetite. Additionally, we assessed the importance of each feature in the DH and NC groups based on the mean decrease in Gini metric (Supplementary Figure S11). Of these taxa, *g_Alistipes*, *g_Alloprevotella*, and *g_Lachnospiraceae_NK4A136_group* were found to be of considerable importance in the DH and NC groups, which is consistent with previous results.

### The alteration of body weight and food intake in mice after FMT treatment

To further verify the effect of altered gut microbiota exposed to HTH environments on appetite, FMT was performed. The result showed that changes in body weight and food intake in the DHF group were significantly lower than those in the NF group (*P* < 0.05) (Fig. 3b and c, Supplementary Table S6), suggesting that the gut microbiome may be the key to appetite inhibition in the HTH environments.

### Altered gut microbiome in mice after FMT treatment

**Fig. 4:**
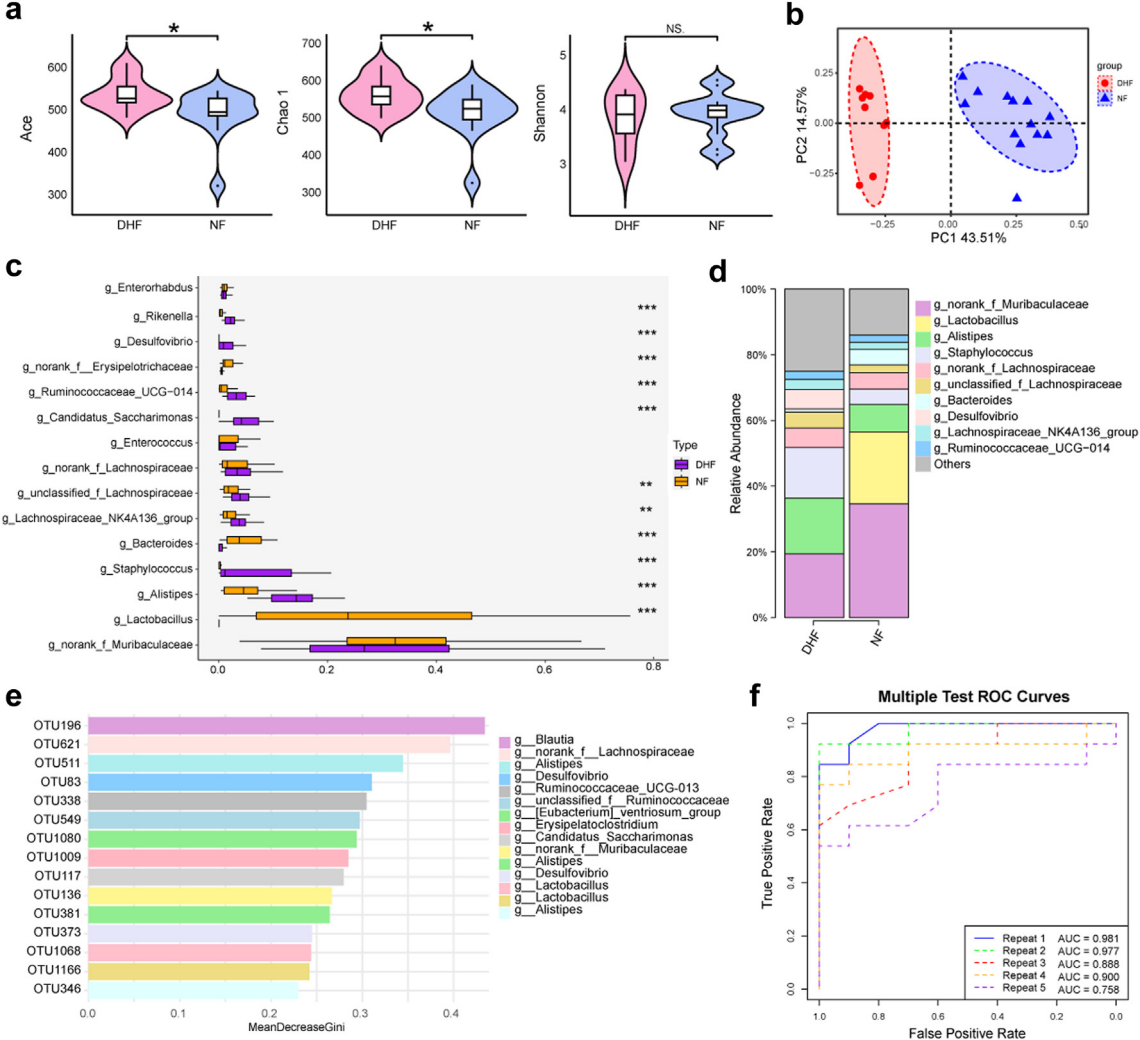
**The alteration of gut microbiome in mice after FMT treatment.(a)** Species diversity differences between the DHF and NF groups (n = 10 and 13, respectively) were estimated by the Ace, Shannon, and Chao1 indices. ∗*P* < 0.05; NS., not significant. **(b)** PCoA plot base of the relative abundance of OTUs by Unweighted UniFrac distance. **(c)** Comparison of relative abundance of bacterial taxa at the genus level between the DHF and NF groups (n = 10 and 13, respectively). The Wilcoxon rank sum test was used to determine the significance between groups. ∗*P* < 0.05, ∗∗*P* < 0.01 vs. the NF group. **(d)** Component proportion of bacteria from fecal content 16S rRNA sequencing data at the genus level between the DHF and NF groups (n = 10 and 13, respectively) (Wilcoxon rank-sum test). **(e)** Random forest (RF) predictor importance of OTUs in the DHF and NF groups (n = 10 and 13, respectively). The legend indicated bacteria at the genus level corresponding to OTU. **(f)** RF classification predictor changes of gut microbiota after FMT treatment. ROC curves showing the accuracy of fitting the test set in RF classification. Statistical significance was evaluated using *P*-values adjusted for multiple comparisons with the False Discovery Rate (FDR) method. In all figures, ∗*P* < 0.05, ∗∗*P* < 0.01, and ∗∗∗*P* < 0.001 denote FDR-adjusted levels of significance.

Next, changes in the gut microbiome of FMT mice were analysed via amplicon sequencing of 16S rRNA genes. There were statistically significant differences between DHF and NF groups in α-diversity, as indicated by the Ace and Chao1 indices (*P* < 0.05), whereas the Shannon index showed no significant difference (Fig. 4a). In β-diversity analysis, both the unweighted PCoA and NMDS plots revealed a separation of the two groups (Fig. 4b; Supplementary Figure S2a). Considerable variability in the gut microbiota across samples was observed in each group. At the phylum level, Bacteroidetes showed a significant difference compared to the NF group (Supplementary Figure S2b). In addition, our data revealed considerable differences in the relative abundance of each bacterium at the family and genus levels across samples from DHF and NF mice. Some differences were also observed in that *f_Rikenellaceae* and *f_Prevotellaceae* were increased in the DHF group, whereas *f_Lactobacillaceae* and *f_Erysipelotrichaceae* were enriched in the NF group (Supplementary Figure S2c and d). DHF mice showed an increment of *g_Staphylococcus*, *g_Lachnospiraceae_NK4A136_group*, and *g_Ruminococcaceae_UCG−014*. In contrast, DHF mice exhibited a loss of *g_Lactobacillus*, *g_Bacteroides*, and *g_norank_f_Erysipelotrichaceae* (Fig. 4c and d, Supplementary Table S11). Taken together, these data indicate alteration of the commensal gut microbiome composition in the DHF group, suggesting dysregulation of the microbial community. To gain a deeper understanding of the relationship between specific members of the gut microbiome and their functions, the importance of features was evaluated in random forest models and screened using the mean decrease Gini indicator. In the model, after FMT, *g_norank_f_Lachnospiraceae*, *g_Alistipes*, and *g_Ruminooccaceae_UCG013* were highly important (Fig. 4e).

### Identification of gut microbial signature for FMT prediction

Next, the random forest method was used to verify the consistency of the gut microbiome between mice after FMT from mice exposed to HTH for 8 weeks and mice exposed to HTH for 8 weeks. First, 995 OTUs were selected from 12 samples from the 8-week DH and NC groups as predictive variables to construct a random forest model (Supplementary Table S12). By utilizing the resampling technique, we determined the optimal model parameters to be mtry = 65. This parameter configuration provides the highest accuracy (approximately 88.89%) and kappa value (approximately 0.75). This indicates that the classifier based on gut microbiota can accurately differentiate between the DH and NC groups. Subsequently, we used samples from the DHF and NF groups, which had undergone FMT, as the test set to validate the model (Supplementary Table S13). This included 23 samples (DHF 10, NF 13). To reduce random errors, we used these 995 OTUs as the test set, repeated the test five times, and evaluated the classification results of the random forest model using ROC analysis. The AUC for these five tests were 0.981, 0.977, 0.888, 0.900, and 0.758, respectively (Fig. 4f). The results showed that even after leaving the HTH environments, appetite suppression could still be observed as long as the structure of the gut microbiota remained unchanged. This provided us with a clue that the gut microbiota might be a key factor affecting appetite.

### Changes in appetite-related hormones in mice exposed to HTH environments

**Fig. 5:**
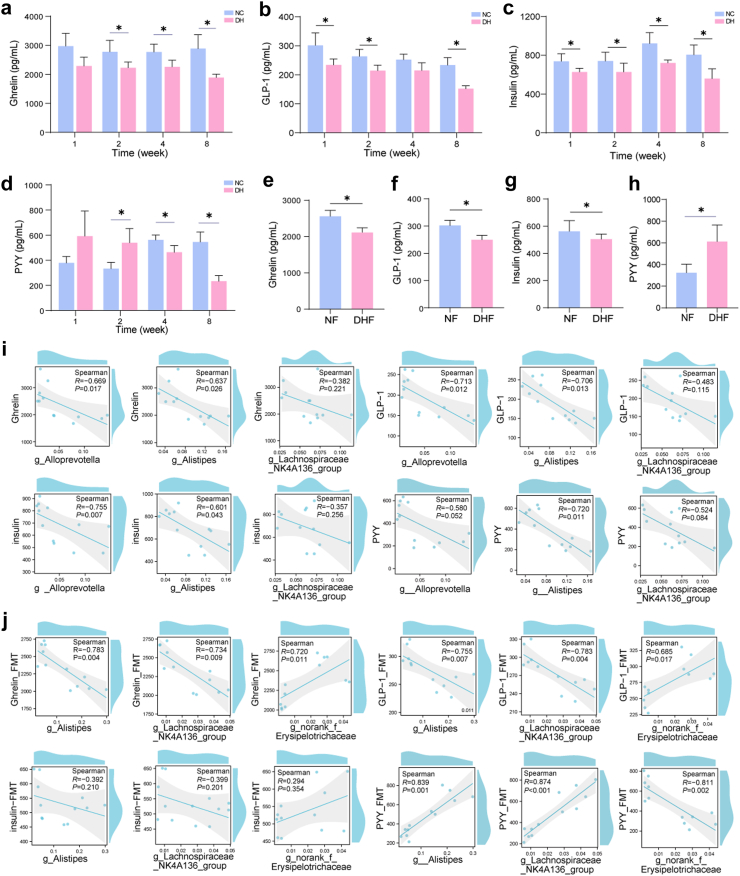
**Effects of HTH conditions on appetite-related hormones in mice.(a–d)** Changes in serum appetite-related hormones in mice exposed to HTH environments at weeks 1, 2, 4, and 8 (n = 6). **(a)** ghrelin; **(b)** GLP-1; **(c)** insulin; **(d)** PYY. **(e–h)** Changes in serum appetite-related hormones in mice after FMT treatment (n = 6). **(e)** ghrelin; **(f)** GLP-1; **(g)** insulin; **(h)** PYY. **(i and j)** Spearman correlation between appetite-related hormones and bacterial taxa at the genus level. **(i)** Mice exposed to HTH environments for 8 weeks; **(j)** mice after FMT treatment.

We next explored the level of appetite-related hormones in mice using a model of mice exposed to the HTH environments and FMT in mice exposed to the HTH environments for 8 weeks. As the exposure time to the HTH environments increased, the expression of appetite-related hormones, including ghrelin, GLP-1, and insulin, significantly decreased in the DH group, and the decrease was the most significant in week 8 (Fig. 5a–c). Notably, PYY levels increased in mice exposed to HTH environments for 1–2 weeks. By the fourth week, it was a significant decrease compared to the NC group (*P* < 0.05), and the decline was more pronounced by week 8 (*P* < 0.05) (Fig. 5d). To further investigate the critical impact of gut microbiota on appetite, environmental factors, such as HTH, were removed but the gut microbiota composition of the DH group was maintained. The results showed that the changes in appetite-related hormones (ghrelin, GLP-1, and insulin) after FMT (DHF group) were consistent with those in the DH group (Fig. 5e–g), while the level of PYY showed an increasing trend (*P* < 0.05) (Fig. 5h). In addition, the differentially expressed bacteria mentioned in the previous section were selected and a correlation analysis was conducted with appetite-related hormones. We found that the levels of ghrelin, GLP-1, insulin, and PYY at week 8 were negatively correlated with *g_Alistipes*, *g_Alloprevotella*, and *g_Lachnospiraceae_NK4A136_group* (Fig. 5i). Moreover, the levels of ghrelin, GLP-1, and insulin in mice after FMT were negatively correlated with *g_Alistipes* and *g_Lachnospiraceae_NK4A136_group* and positively correlated with *g_norank_f_Erysipelotrichaceae*, whereas the level of PYY was positively correlated with *g_Alistipes* and *g_Lachnospiraceae_NK4A136_group* and negatively correlated with *g_norank_f_Erysipelotrichaceae* (Fig. 5j). We demonstrated a significant alteration in gut microbiota-associated appetite-related hormones. These findings and our current findings indicate that the gut microbiome plays a key role in the regulation of appetite and appetite-related hormones.

### Causal relationship between microbial taxa and appetite

**Fig. 6:**
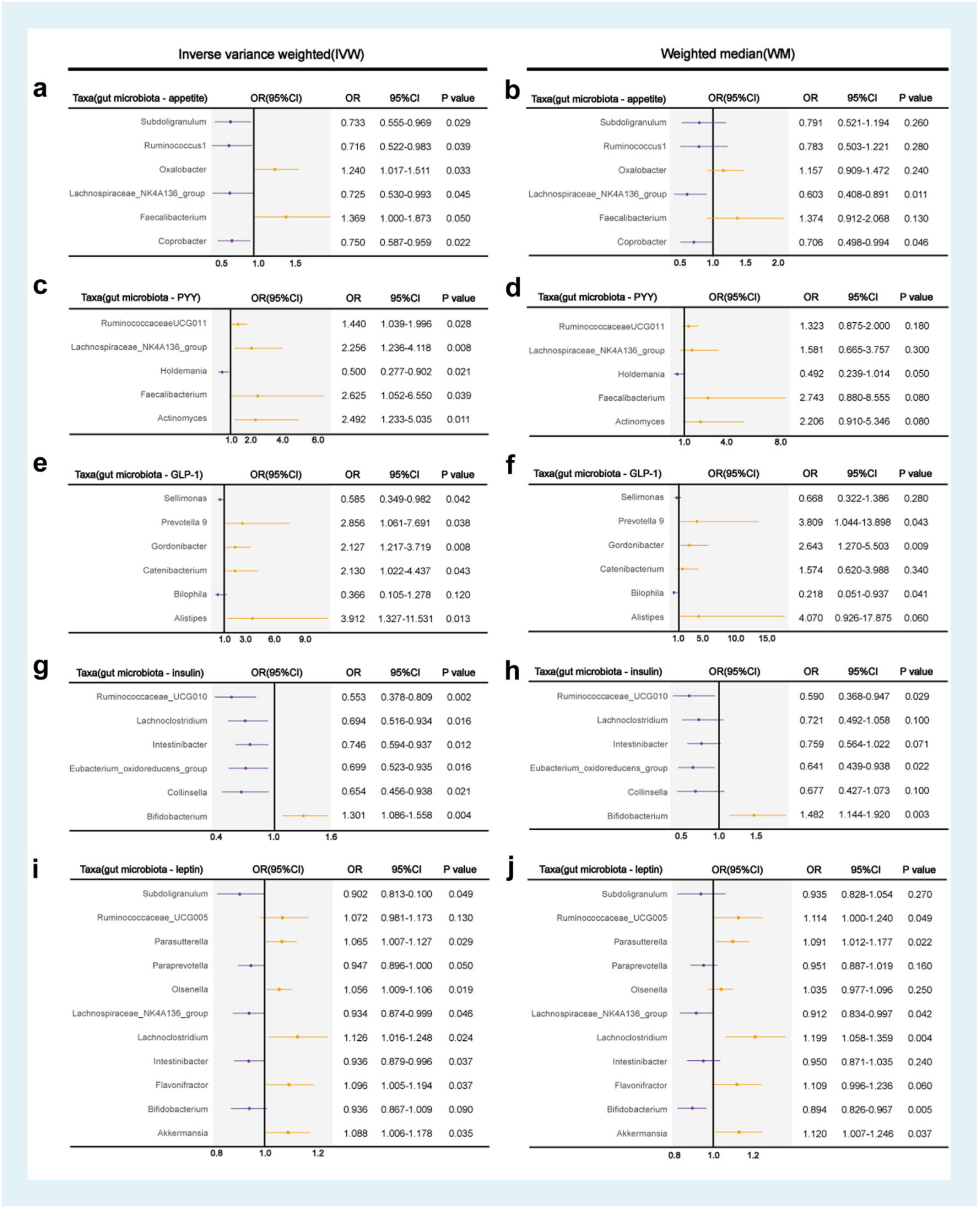
**Mendelian randomization (MR) analysis on the causal effect of the gut microbiota on appetite and appetite-related hormones.(a and b)** The forest plot shows the causal estimates between microbial taxa at the genus level and appetite. The OR and 95% CI were obtained using the inverse variance weighted and weighted median method. **(a)** The inverse variance weighted method; **(b)** weighted median method. **(c–j)** The forest plot shows the causal estimates between microbial taxa at the genus level and appetite-related hormones. **(c and d)** PYY; **(e and f)** GLP-1. **(g and h)** Insulin; **(i and j)** leptin. The OR and 95% CI were obtained using the inverse variance weighted and weighted median method. **(c, e, g, i)** the inverse variance weighted method; **(d, f, h, j)** weighted median method. OR, odds ratio; CI, confidence interval; PYY, peptide YY; GLP-1, glucagon-like peptide-1. Data Sources: Gut microbiota (MiBioGen); Appetite (UK Biobank); PYY (Experimental Factor Ontology); Leptin (IEU OpenGWAS); GLP-1 and Insulin (GWAS Catalog). Statistical significance was evaluated using *P*-values adjusted for multiple comparisons with the False Discovery Rate (FDR) method.

Based on the idea^12^ that gut microbial composition is a key factor in regulating appetite, we utilised MR to explore the impact of the gut microbiome on appetite. To validate this association, we employed the inverse variance-weighted method, MR-Egger method, and weighted median MR sensitivity analysis to identify the microbial taxa associated with appetite (Fig. 6a and b, Supplementary Tables S1 and S21), and evaluated the reliability of the results through multiple sensitivity analyses (Supplementary Figure S3 and Table S26). Among these taxa, *g_Lachnospiraceae_NK4A136_group* showed a significant results, with an OR of 0.725 in the IVW method and a 95% confidence interval (CI) of 0.530–0.993 (*P* = 0.045) (Fig. 6a). In the weighted median method, the OR was 0.603, with a 95% CI of 0.408–0.891 (*P* = 0.011), and the *P* value in the MR-Egger method was 0.06 (Fig. 6b, Supplementary Tables S1 and S21). These results indicate that *g_Lachnospiraceae_NK4A136_group* had a negative causal relationship with appetite. Additionally, several other microbial taxa have been suggested to exert causal effects on appetite. Specifically, *g_Coprobacter* (OR = 0.750, 95% CI = 0.587–0.959, *P* = 0.022), *g_Faecalibacterium* (OR = 1.369, 95% CI = 1.000–1.873, *P* = 0.05), *g_Oxalobacter* (OR = 1.240, 95% CI = 1.017–1.511, *P* = 0.033), *g_Ruminococcus 1* (OR = 0.716, 95% CI = 0.522–0.983, *P* = 0.039), and *g_Subdoligranulum* (OR = 0.733, 95% CI = 0.555–0.969, *P* = 0.029) may also have potential causal effects on appetite in the IVW method (Fig. 6a). The multi-pleiotropy test using the MR-Egger intercept indicated that the *P*-value of the intercept was >0.05, suggesting that directional pleiotropy is unlikely to occur in these results (Supplementary Table S1). The F-statistics for all the microbial taxa were greater than 10. Notably, the gut microbes that showed a significant causal relationship with appetite were compared using 16S rRNA gene sequencing. We found that the increased quantity of *g_Lachnospiraceae_NK4A136_group* was consistent with appetite suppression, and *g_Ruminococcaceae* displayed a similar trend. This provides strong evidence that these two bacterial taxa may be significant factors influencing appetite.

### Causal relationship between gut microbiota and appetite hormones

To further explore the potential causal relationship between gut microbiota and appetite hormones, we used MR analysis of the intestinal flora. We selected appetite-related hormones, leptin, PYY, GLP-1, and insulin and further analysed the specific gut microbes that were significantly associated with these hormones (Fig. 6c–j, Supplementary Tables S22–S25), and evaluated the reliability of the results through multiple sensitivity analyses (Supplementary Figures S4 and S5 and Tables S27–S29). The results showed a significant causal relationship between *g_Lachnospiraceae_NK4A136_group* and leptin (OR = 0.934, 95% CI = 0.874–0.999, *P* = 0.046) and PYY (OR = 2.256, 95% CI = 1.236–4.118, *P* = 0.008) (Fig. 6c and i), suggesting a negative causal relationship between *g_Lachnospiraceae_NK4A136_group* and leptin and a strong positive causal relationship between *g_Lachnospiraceae_NK4A136_group* and PYY. A significant causal relationship was observed between *g_Alistipes* and GLP-1 (OR = 3.912, 95% CI = 1.327–11.531, *P* = 0.013; Fig. 6e). Ruminococcus species showed strong causal relationships across various appetite-related hormones, specifically in a positive causal relationship between *g_Ruminococcaceae_UCG-011* and PYY (OR = 1.440, 95% CI = 1.039–1.996, *P* = 0.028, Fig. 6c), a negative causal relationship between *g_Ruminococcaceae_UCG-010* and insulin (OR = 0.553, 95% CI = 0.378–0.809, *P* = 0.002, Fig. 6g), and a positive causal relationship *g_Ruminococcaceae_UCG-005* and leptin (OR = 1.072, 95% CI = 0.981–1.173, *P* = 0.130, Fig. 6i). Considering potential heterogeneity, we also employed a weighted median estimator as a robust method. The causal relationship between *g_Lachnospiraceae_NK4A136_group* and leptin remained significant (OR = 0.912, 95% CI = 0.834–0.997, *P* = 0.042; Fig. 6j), showing a negative correlation. The causal relationship between *g_Ruminococcaceae_UCG-010* and insulin was also significant (OR = 0.590, 95% CI = 0.368–0.947, *P* = 0.029; Fig. 6h) and negatively correlated. *g_Ruminococcaceae_UCG-005* (*P* = 0.049, Fig. 6j) with leptin, and *g_Alistipes* with GLP-1 (*P* = 0.060, Fig. 6f) indicated a strong causal relationship between them. Combined with previous correlation analyses, we also preliminarily determined that there was a positive correlation between *g_Lachnospiraceae_NK4A136_group* and PYY and that there might be a negative correlation between *g_Alistipes* and GLP-1. However, further studies are required to test this hypothesis. Additionally, we used the simple and weighted modes for causal effect estimation of the gut microbiota with appetite and appetite-related hormones (data shown in Supplementary Tables S1–S5).

## Discussion

In the present study, in addition to conducting a longitudinal investigation into whether HTH environments suppress the appetite of mice, 16S rRNA sequencing method was used in a FMT experiment. In our study, we demonstrated that a reduction in appetite was associated with changes in the composition and function of the gut microbiome, as well as moderately decreased levels of diversity. Dysbiosis in the gut microbiome was observed by week 2 in the HTH environments. By week 8, there were complete changes observed in the composition and diversity of the gut microbial communities. We found the gut microbiome in the DH group exhibited significant changes in the overall microbial diversity. After FMT, the DHF group continued to exhibit a suppressed appetite even in the normal environments, whereas the NF group displayed higher body weight gain and food consumption than the DHF group. Our results emphasise the mediating role of the gut microbiome on appetite in HTH environments, which was further confirmed through validation experiments. We also established a random forest classifier to assess the modelling of FMT and repeatedly tested the validation set to demonstrate that the post-FMT gut microbial composition was highly similar to that of the DH and NC groups at week 8 (AUC = 0.981). Subsequently, we conducted a feature importance analysis (based on the MeanDecreaseGini) on the gut microbiota of mice exposed to HTH conditions at week 8 and after FMT, and in combination with differential bacteria from the DH and NC groups. The results showed that under HTH conditions, bacterial taxa, such as *g_Ruminococcaceae_UCG-014*, *g_Alistipes*, and *g_Lachnospiraceae_NK4A136_group* underwent substantial changes. Specifically, increases in *g_Alistipes*, *g_Lachnospiraceae_NK4A136_group*, and *g_Ruminococcaceae_UCG-014* were characteristic of the changes in the gut microbiome under conditions of suppressed appetite. Following this, we will employ MR analysis to explore the causal relationship between these differentially abundant gut microbes and appetite and to verify whether changes in *g_Ruminococcaceae_UCG-014*, *g_Alistipes*, and *g_Lachnospiraceae_NK4A136_group* are associated with appetite-regulating hormones.

Hormones secreted by the gastrointestinal tract serve as regulators of appearance by mediating hunger and satiety.^53^^,^^54^ Ghrelin, the only known orexigenic peripheral peptide, has been extensively studied for its precise role in appetite regulation.^55^^,^^56^ We found that the expression of ghrelin was significantly lower in the DH and DHF groups than that in the NC or NF groups, suggesting an impact of the HTH environments on appetite. Peptide YY (PYY; also known as peptide tyrosine-tyrosine), an anorexigenic gastrointestinal hormone, plays a significant role in appetite regulation, particularly satiety and meal termination.^57^ In our study, PYY showed an increasing trend in mice exposed to an HTH environments for 1–2 weeks, and an increasing trend after FMT (the DHF group). GLP-1 and insulin levels were significantly low in the DH and DHF groups. Insulin plays crucial roles in appetite and hunger.^58^ Research has found that reducing insulin levels can effectively control hunger and appetite, which is consistent with our results. Although GLP-1 is an anorexic hormone, it promotes insulin secretion.^58^^,^^59^ We found that in mice exposed to humid and hot environments, serum GLP-1 levels showed a continuous downward trend, which may be closely related to a reduction in insulin.^60^

In the current large-scale and comprehensive MR study, six microbial taxa, including *g_Lachnospiraceae_NK4A136_group*, that had a causal relationship with the risk of poor appetite or overeating, were identified. The TS-MR results suggest that certain gut microbiota are crucial influencers of appetite-regulating hormones, among which *g_Lachnospiraceae_NK4A136_group* might mediate the appetite-suppressing effects of PYY. Our analysis of 16SrRNA sequencing and MR data regarding the relationships among the gut microbiome, appetite hormones, and poor appetite provides evidence for a causal link between the gut microbiome and poor appetite, as well as the mediating role of appetite hormones.

**Fig. 7:**
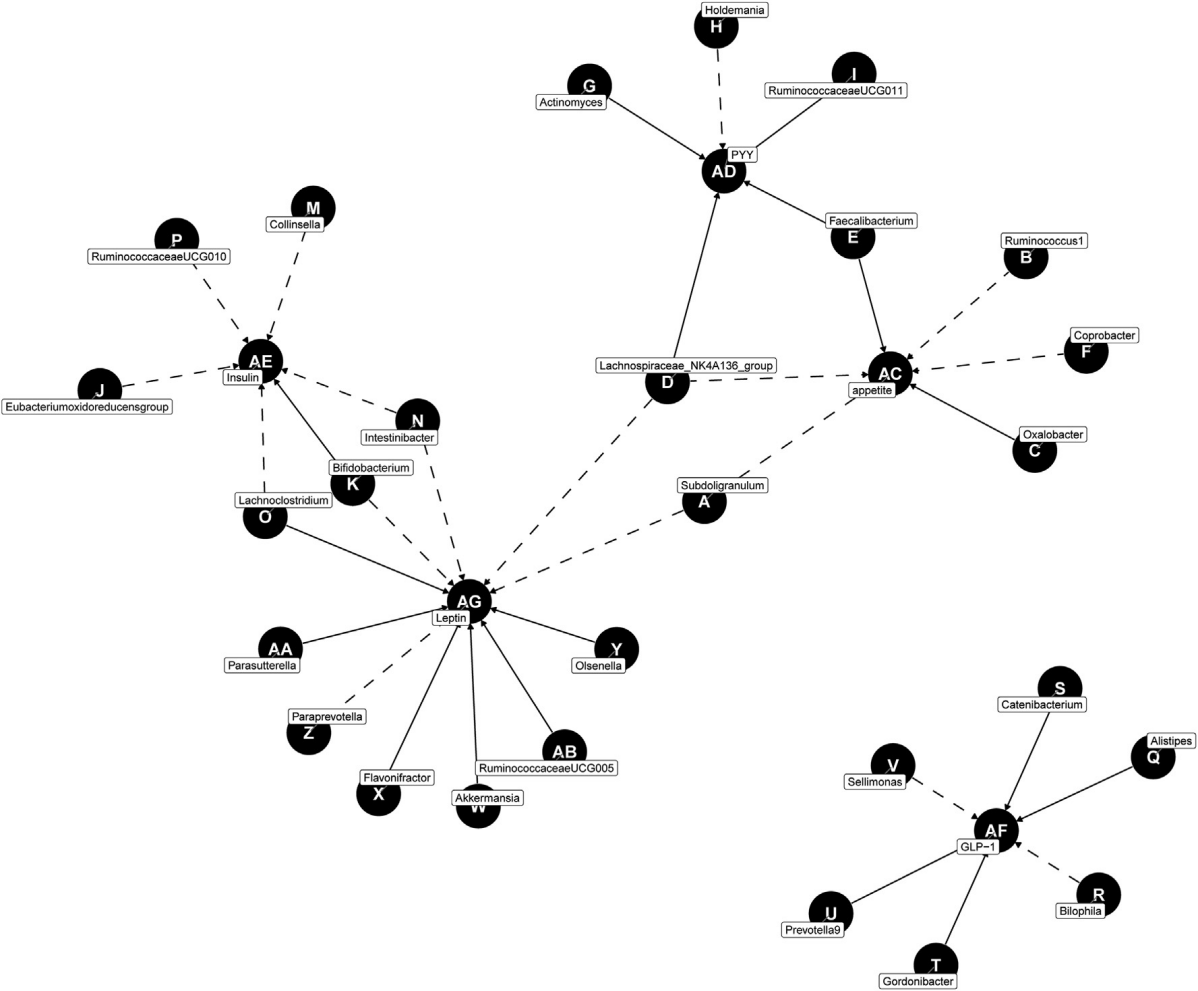
**A directed acyclic graph (DAG) of the significant causal relationship between gut microbiota and appetite/appetite hormones.** This figure shows the direct causal relationship between the gut microbiota and related appetite factors, with the parent representing the starting point of the causal relationship and the child representing the endpoint of the causal relationship. Dashed lines, OR less than 1; solid lines, OR greater than 1.

Through MR analysis, we summarised the significant causal relationship between gut microbiota and appetite- or appetite-related hormones, and created a directed acyclic graph to visualize their relationship (Fig. 7). We found *g_Lachnospiraceae_NK4A136_group* is a major taxon within the *Lachnospiraceae* genus of the human gut microbiota, and our study revealed a causal relationship between it and poor appetite. In the mediation MR analysis, the species *g_Lachnospiraceae_NK4A136_group* was found to cause an increase in PYY levels, which was subsequently correlated with an increased risk of poor appetite. A study on obesity and gut microbiome dysbiosis suggested that transplantation of microbiota altered by polyamines could prevent obesity.^61^ These changes may be partially driven by *g_Lachnospiraceae_NK4A136_group*, which produce SCFAs.^62^ The abundance of this bacterium was reduced in individuals with obesity but increased in the presence of polyamines. *g_Lachnospiraceae_NK4A136_group* is considered a crucial bacterium in counteracting obesity,^63^^,^^64^ and plays an important defensive role in several gut-related diseases, such as DSS-induced ulcerative colitis in mice, diabetes, and non-alcoholic fatty liver disease.^65^, ^66^, ^67^ Moreover, changes in *g_Lachnospiraceae _NK4A136_group* were positively correlated with changes in adherence to the Mediterranean diet in dietary balance studies.^68^^,^^69^

Furthermore, *g_Alistipes* was more abundant in the DH group than in the NC group. MR analysis revealed a causal relationship between *g_Alistipes* and GLP-1. Finally, based on correlation analysis, we speculated that *g_Alistipes* might regulate GLP-1 levels, subsequently leading to an increased risk of poor appetite. *g_Alistipes* is a relatively new genus, primarily isolated from clinical medical samples. Although its abundance is lower than that of other genera within the phylum Bacteroidetes, it is strongly associated with dysbiosis and diseases.^70^ Meanwhile, *g_Ruminococcaceae_UCG-014*, which had a notably increased abundance in the DH and DHF groups, showed in the MR analysis that its counterparts, *g_Ruminococcaceae_UCG-005*, *g_Ruminococcaceae_UCG-010*, and *g_Ruminococcaceae_UCG-011*, had causal relationships with leptin, insulin, and PYY, respectively. Hu et al. indicated that a reduction in *g_Ruminococcaceae_UCG-014*, *g_Lactobacillus*, and a few other genera was associated with a decrease in obesity parameters.^71^^,^^72^ We believe that *g_Ruminococcaceae_UCG-014*, *g_Alistipes*, and *g_Lachnospiraceae_NK4A136_group* mediate appetite-related hormones, which are likely the key factors causing appetite suppression in the HTH environments.

However, the absence of clinical research to corroborate our findings from the mouse model is a significant limitation. The complexity of the human gut microbiota and its interaction with diverse environmental factors may not be fully replicated in a controlled laboratory setting. Moving forward, we hope to extend our research to include clinical studies that will allow us to explore the implications of our findings in humans. We aim to investigate how HTH environments affect the gut microbiota and appetite in clinical settings, thereby enhancing the translational value of our work. This future direction will also enable us to potentially develop microbiota-based interventions for appetite control and metabolic health in humans exposed to similar environmental conditions.

In summary, this study demonstrates changes in gut microbiota in mice caused by HTH are associated with decreased appetite. Subsequently, MR analysis was used to verify the causal relationship between the above-mentioned changes in intestinal bacteria and appetite- or appetite-related hormones, and the results were highly consistent with those of the animal experiments. These well-defined changes may provide important references for the clinical application and development of probiotics for the treatment of eating disorders and weight control.

## Contributors

MJL, ZRH, and YHZ performed animal experiments. YLW, XRF, and MJL data curation, formal analysis and data verification. YLW and XRF analysed 16S RNA-sequencing data and Mendelian randomisation, verified the data and prepared the manuscript. SC and HL supervision. HL project administration, resources and funding acquisition. All authors have read and approved the final version of the manuscript.

## Data sharing statement

Primary experimental data will be shared upon personal request by the corresponding authors. 16S rRNA sequencing data were deposited in the BioProject database (Accession numbers are PRJNA1032397 and PRJNA1032695), and are publicly available.

## Declaration of interests

Authors have no conflicts of interest to report.

## Appendix A Supplementary data

Supplementary Tables S6–S29

Study Protocol

Reagent Validation
