# Supplementary material for: Gut microbiota associated with appetite suppression in high-temperature and high-humidity environments
Source: eBioMedicine. 2023 Dec 16;99:104918. doi: 10.1016/j.ebiom.2023.104918 (PMC10765014; doi:10.1016/j.ebiom.2023.104918)
Supplement: Supplementary Figures S1–S5 and Tables S1–S5 [file mmc1.docx]

**Supplemental Information**

**Gut microbiota associated with appetite suppression in high-temperature and high-humidity environments**


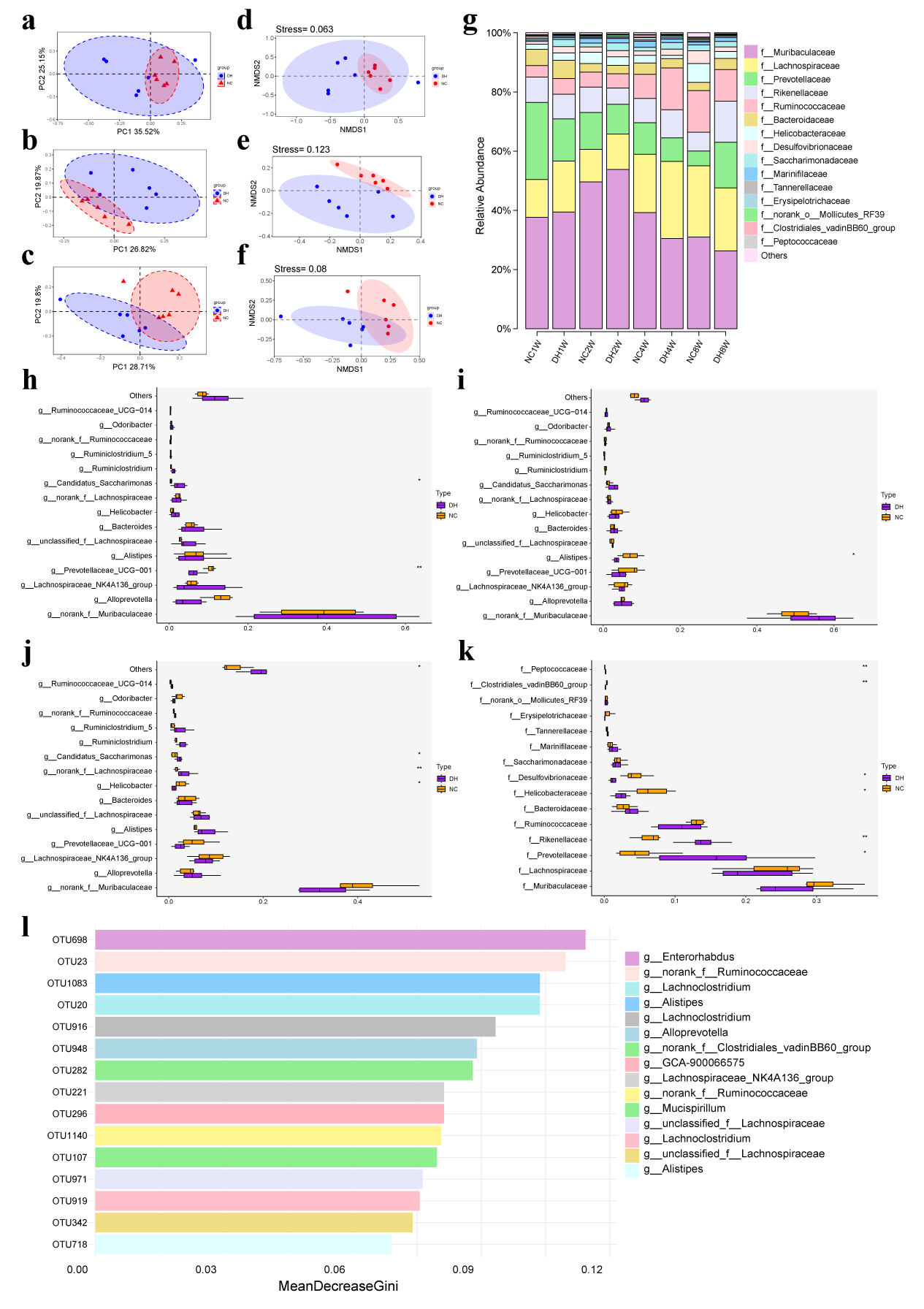


**Fig S1:** **The alteration of gut microbiome in mice exposed to HTH environments. (a-c)** PCoA plot base of the relative abundance of OTUs by Unweighted UniFrac distance between the DH and NC groups at weeks 1, 2, and 4 (n=6 for all groups). **(e-f)** NMDS plot of the relative abundance of OTUs between the DH and NC groups at weeks 1, 2, and 4. **(g)** Component proportion of bacteria from fecal content 16S rDNA sequencing data at the family level (top 15) between the DH and NC groups (Wilcoxon rank-sum test). **(h-j)** Comparison of relative abundance of bacterial taxa at the genus level between the DH and NC groups at at weeks 1, 2, and 4. The Wilcoxon rank sum test was used to determine the signifcance between groups. *P < 0.05, **P < 0.01 vs. the NC group. **(k)** Comparison of relative abundance of bacterial taxa at the family level between the DH and NC groups at week 8. The Wilcoxon rank sum test was used to determine the signifcance between groups. *P < 0.05, **P < 0.01 vs. the NC group. **(l)** Random forest (RF) mean predictor importance of OTUs in the DH and NC group. The legend indicated bacteria at the genus level corresponding to OTU. Statistical significance was evaluated using p-values adjusted for multiple comparisons with the False Discovery Rate (FDR) method. In all figures, *P < 0.05, and **P < 0.01 denote FDR-adjusted levels of significance.

~~
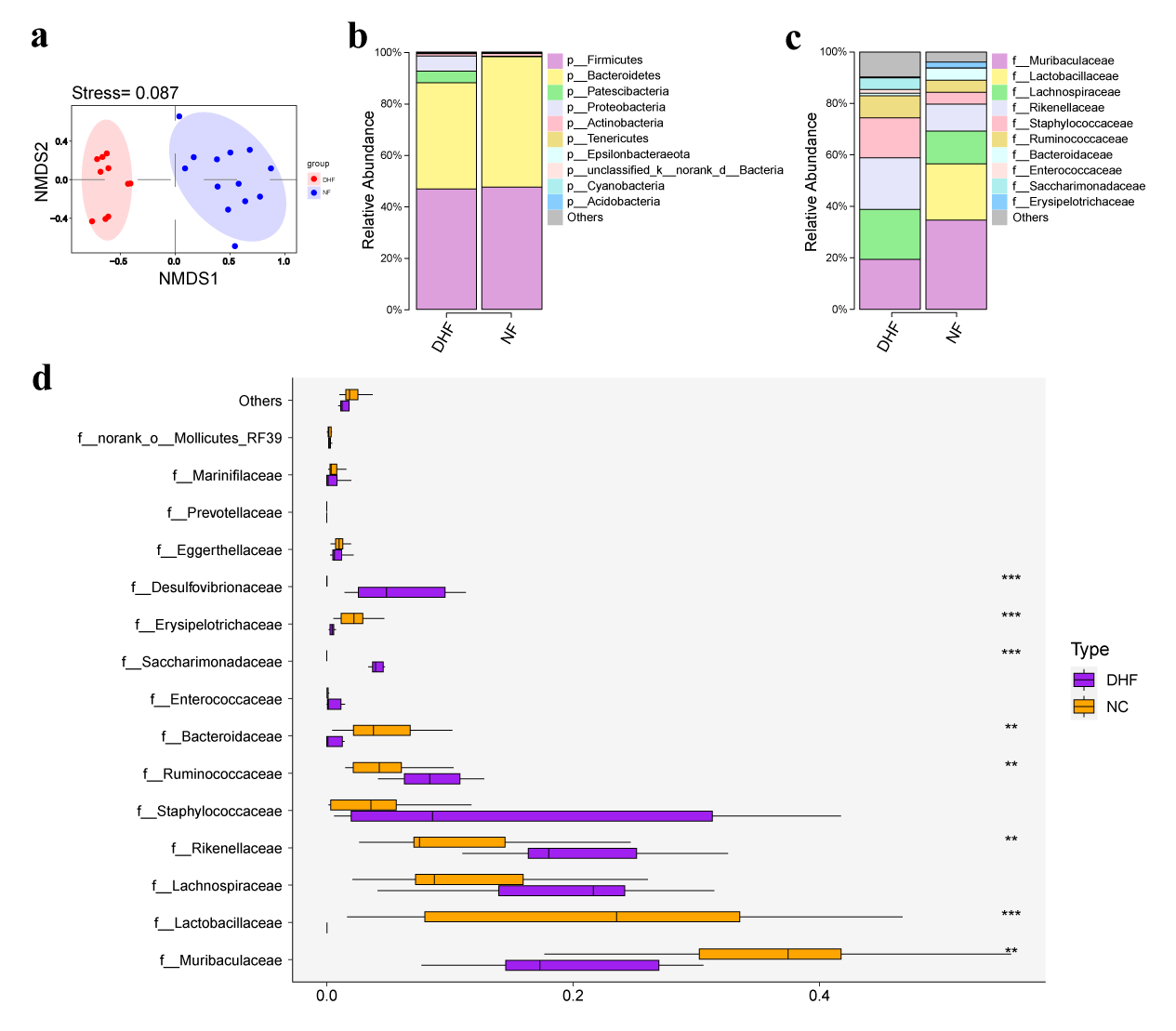
~~

**Fig. S2.** **The alteration of gut microbiome after FMT treatment.** **(a)** NMDS plot of the relative abundance of OTUs between the DHF and NF groups. **(b-c)** Component proportion of bacteria from fecal content 16S rDNA sequencing data at the phylum and family levels between the DHF and NF groups, respectively (Wilcoxon rank-sum test). **(d)** Comparison of relative abundance of bacterial taxa at the family level between the DHF and NF groups. The Wilcoxon rank sum test was used to determine the signifcance between groups. *P < 0.05, **P < 0.01 vs. the NF group. Statistical significance was evaluated using p-values adjusted for multiple comparisons with the False Discovery Rate (FDR) method. In all figures, *P < 0.05, **P < 0.01, and ***P < 0.001 denote FDR-adjusted levels of significance.


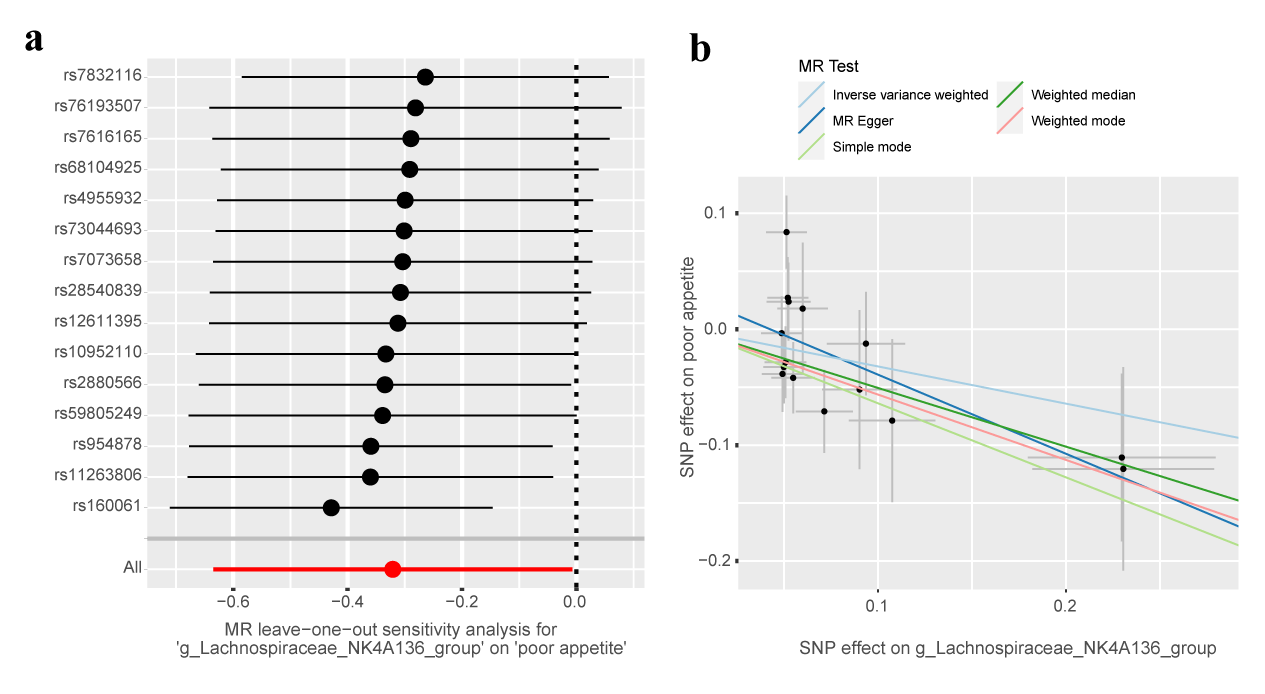


**Fig. S3. Leave-one-out sensitivity analysis and pleiotropy test for significant MR linkages. (a)** Forest plots of MR leave-one-out sensitivity results (IVW method) for significant MR linkages in gut microbiota and appetite. Dots represent the estimated effect size. Bars represent 95% confidence intervals. The x-axis indicates effect size of MR. **(b)** Correlation scatter plot of MR analysis in gut microbiota and appetite.


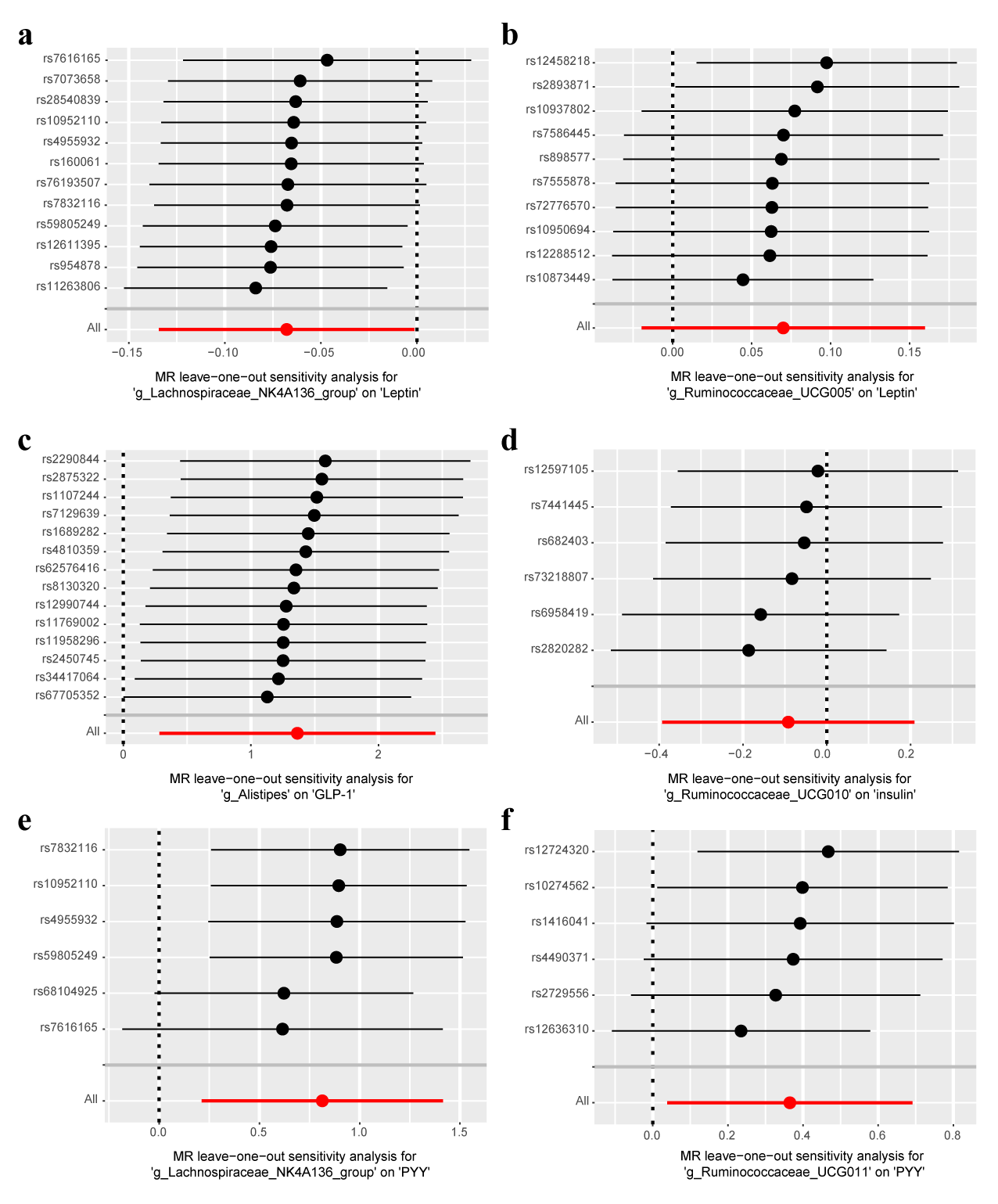


**Fig. S4. Leave-one-out sensitivity analysis for significant MR linkages.** Forest plots of MR leave-one-out sensitivity results (IVW method) for significant MR linkages in gut microbiota and appetite-related hormones. Dots represent the estimated effect size. Bars represent 95% confidence intervals. The x-axis indicates effect size of MR.


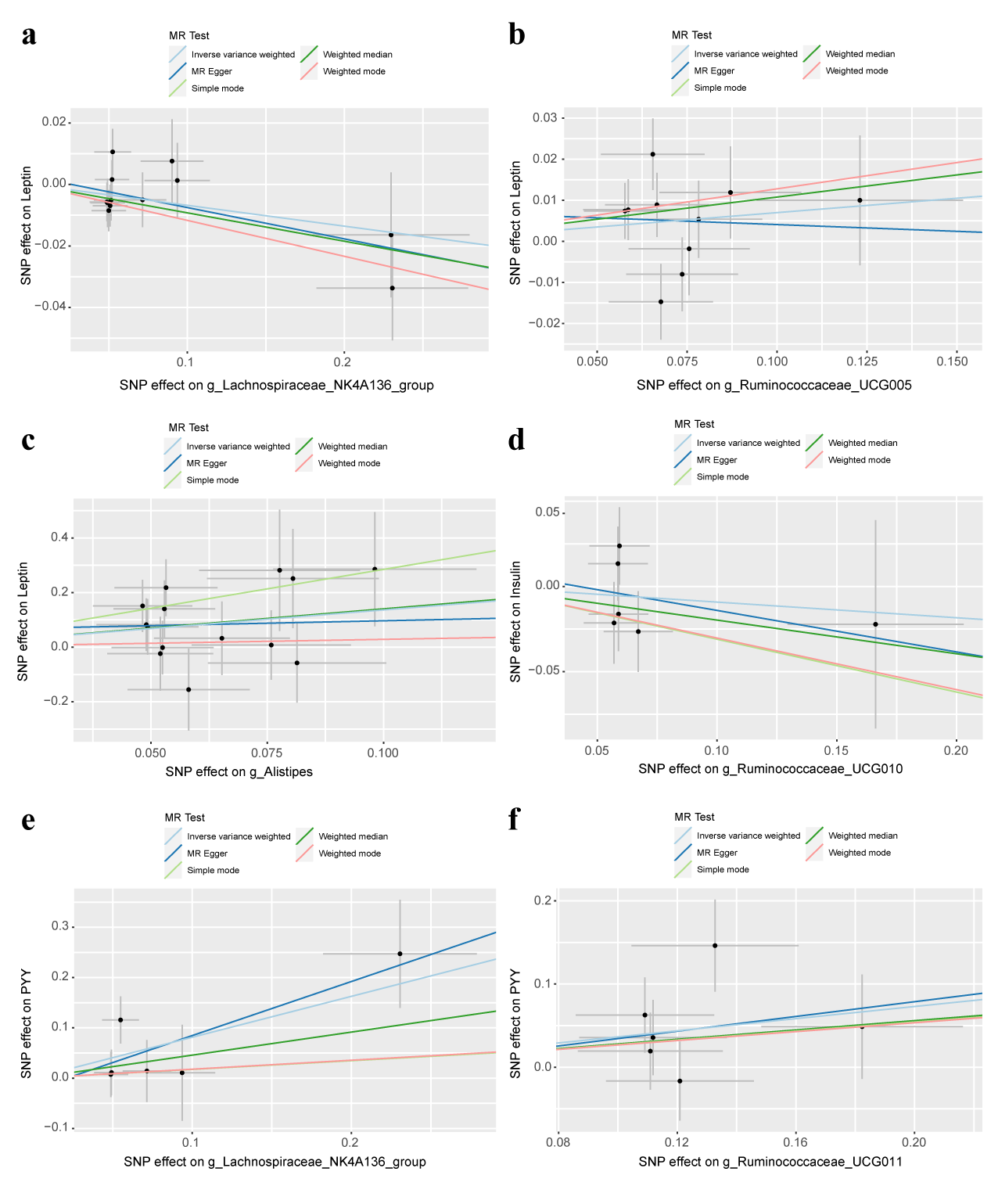


**Fig. S5. Pleiotropy test for significant MR linkages.** Correlation scatter plot of MR Pleiotropy test results (IVW method) for significant MR linkages in gut microbiota and appetite-related hormones.


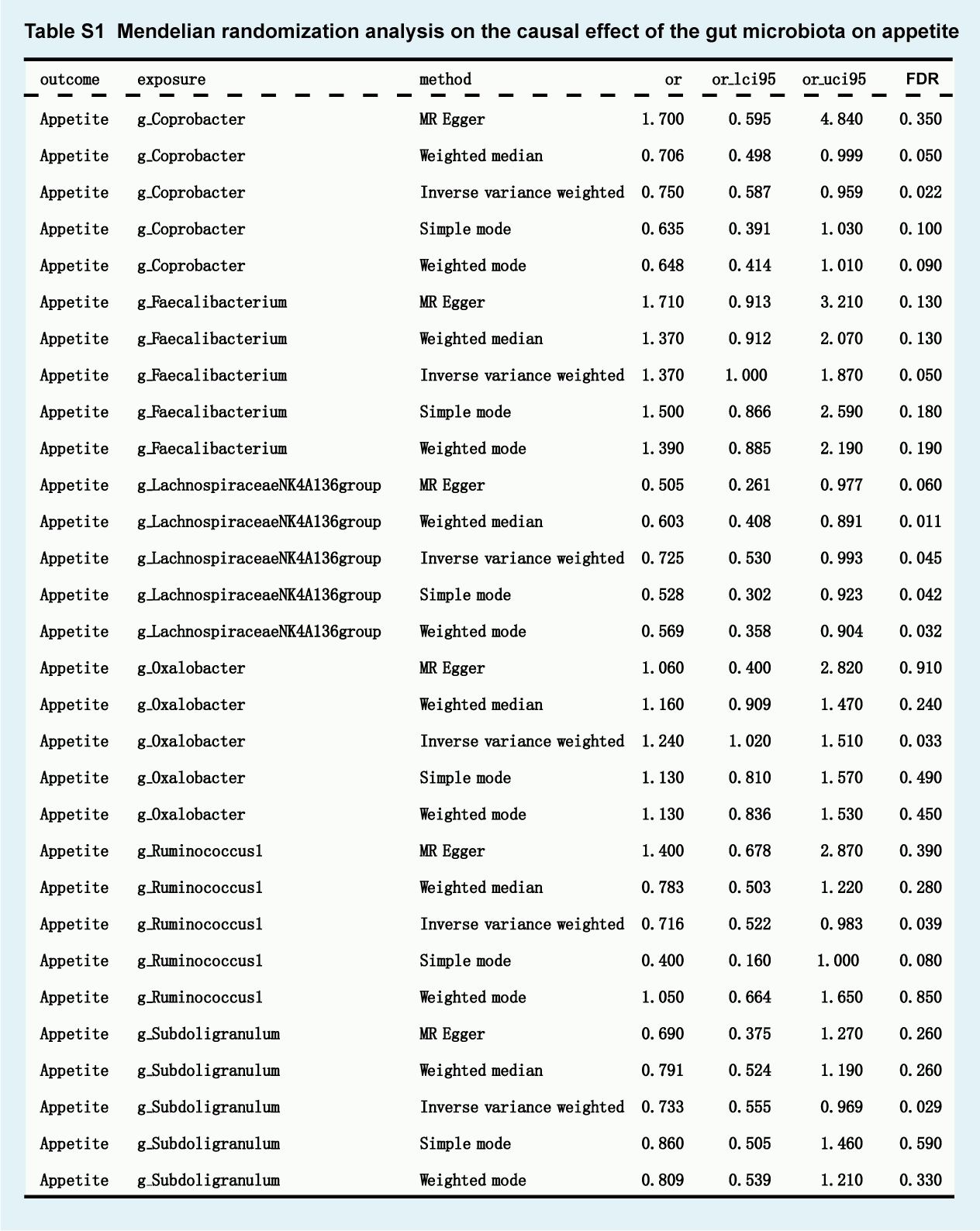


**Table S1. Mendelian Randomization analysis on the causal effect of gut microbiota on appetite**. Inverse variance weighted, Weight median, MR Egger, Simple mode, and Weight mode were used to identify the microbial taxa associated with appetite. Or, or_lci95, or_uci95 and FDR were calculated for the respective method of MR analysis. Or, odds ratio; or_lci95, odds ratio_low confidence interval 95; or_uci95, odds ratio_up confidence interval 95.


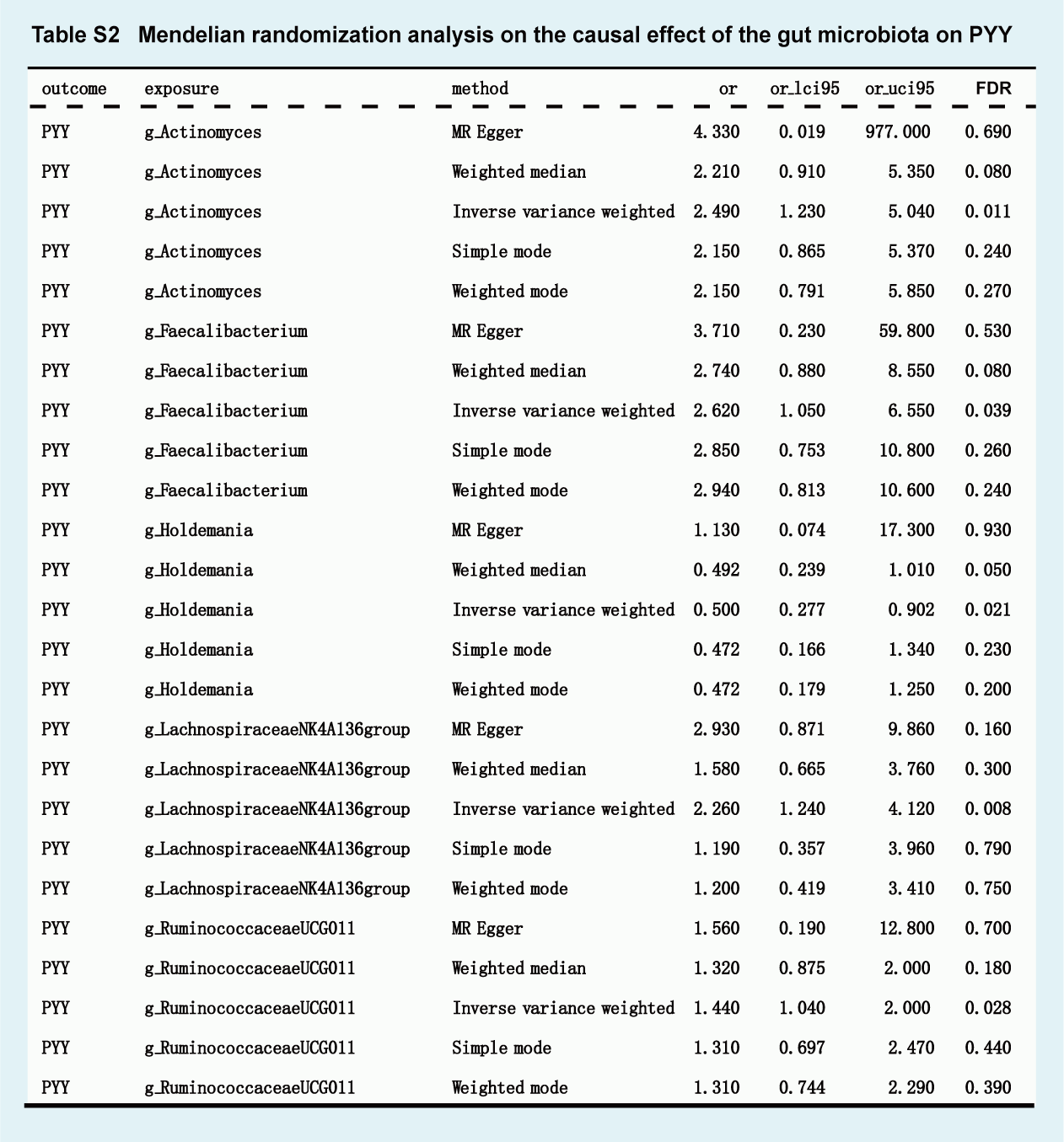


**Table S2. Mendelian Randomization analysis on the causal effect of gut microbiota on PYY**. Inverse variance weighted, Weight median, MR Egger, Simple mode, and Weight mode were used to identify the microbial taxa associated with appetite. Or, or_lci95, or_uci95 and FDR were calculated for the respective method of MR analysis. Or, odds ratio; or_lci95, odds ratio_low confidence interval 95; or_uci95, odds ratio_up confidence interval 95; PYY, peptide YY.


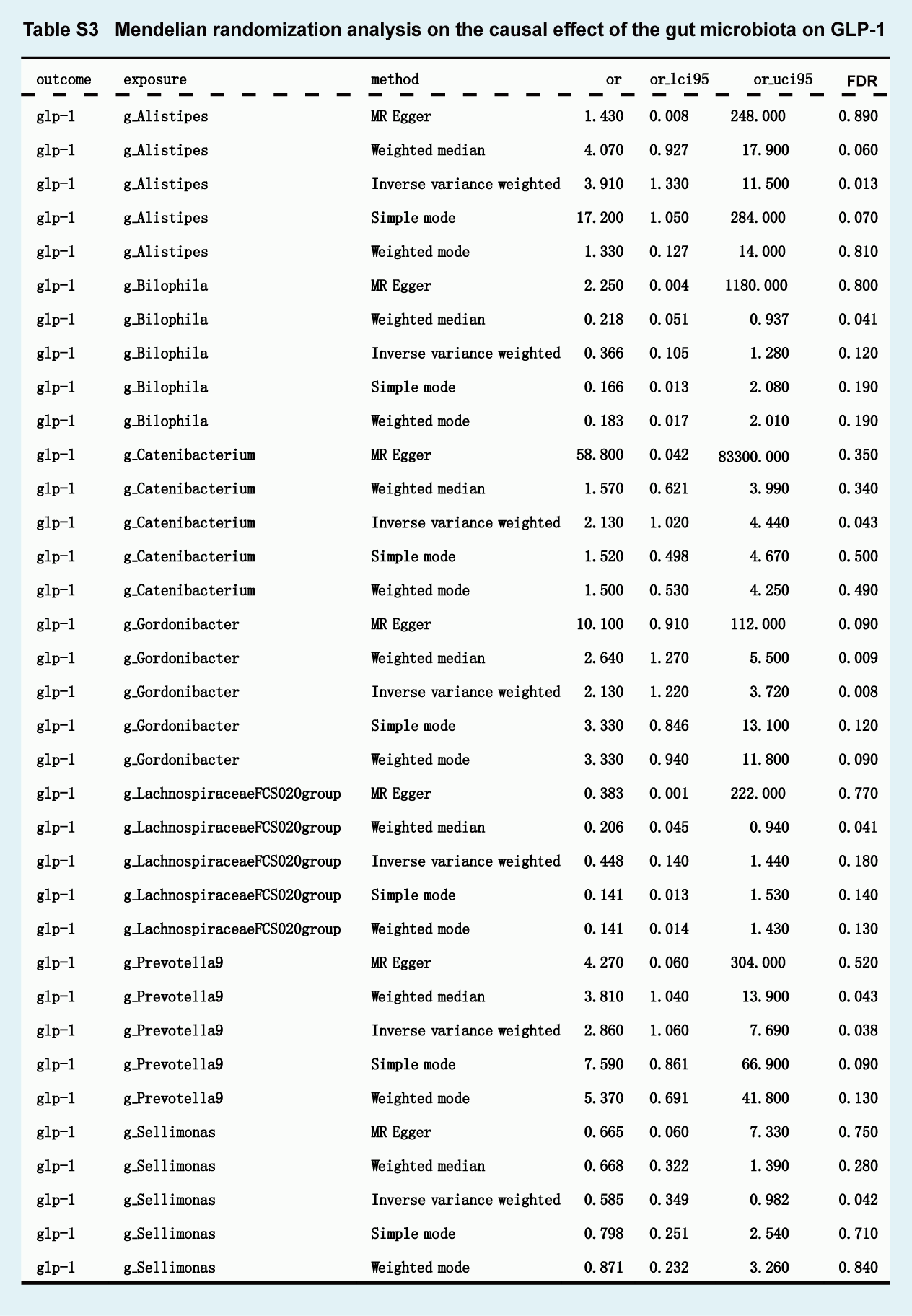


**Table S3. Mendelian Randomization analysis on the causal effect of gut microbiota on GLP-1**. Inverse variance weighted, Weight median, MR Egger, Simple mode, and Weight mode were used to identify the microbial taxa associated with appetite. Or, or_lci95, or_uci95 and FDR were calculated for the respective method of MR analysis. Or, odds ratio; or_lci95, odds ratio_low confidence interval 95; or_uci95, odds ratio_up confidence interval 95; GLP-1, glucagon-like peptide-1.


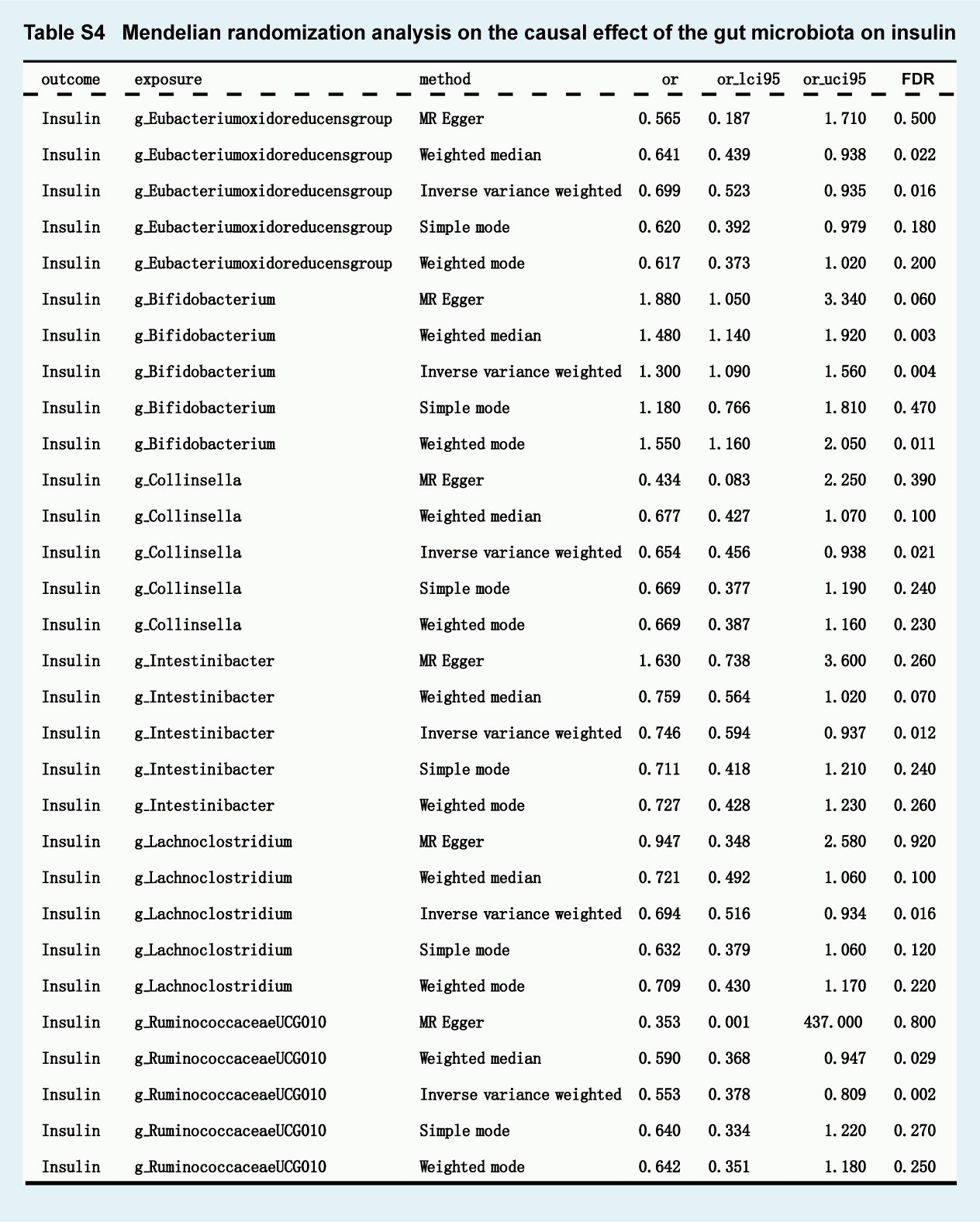


**Table S4. Mendelian Randomization analysis on the causal effect of gut microbiota on insulin**. Inverse variance weighted, Weight median, MR Egger, Simple mode, and Weight mode were used to identify the microbial taxa associated with appetite. Or, or_lci95, or_uci95 and FDR were calculated for the respective method of MR analysis. Or, odds ratio; or_lci95, odds ratio_low confidence interval 95; or_uci95, odds ratio_up confidence interval 95.


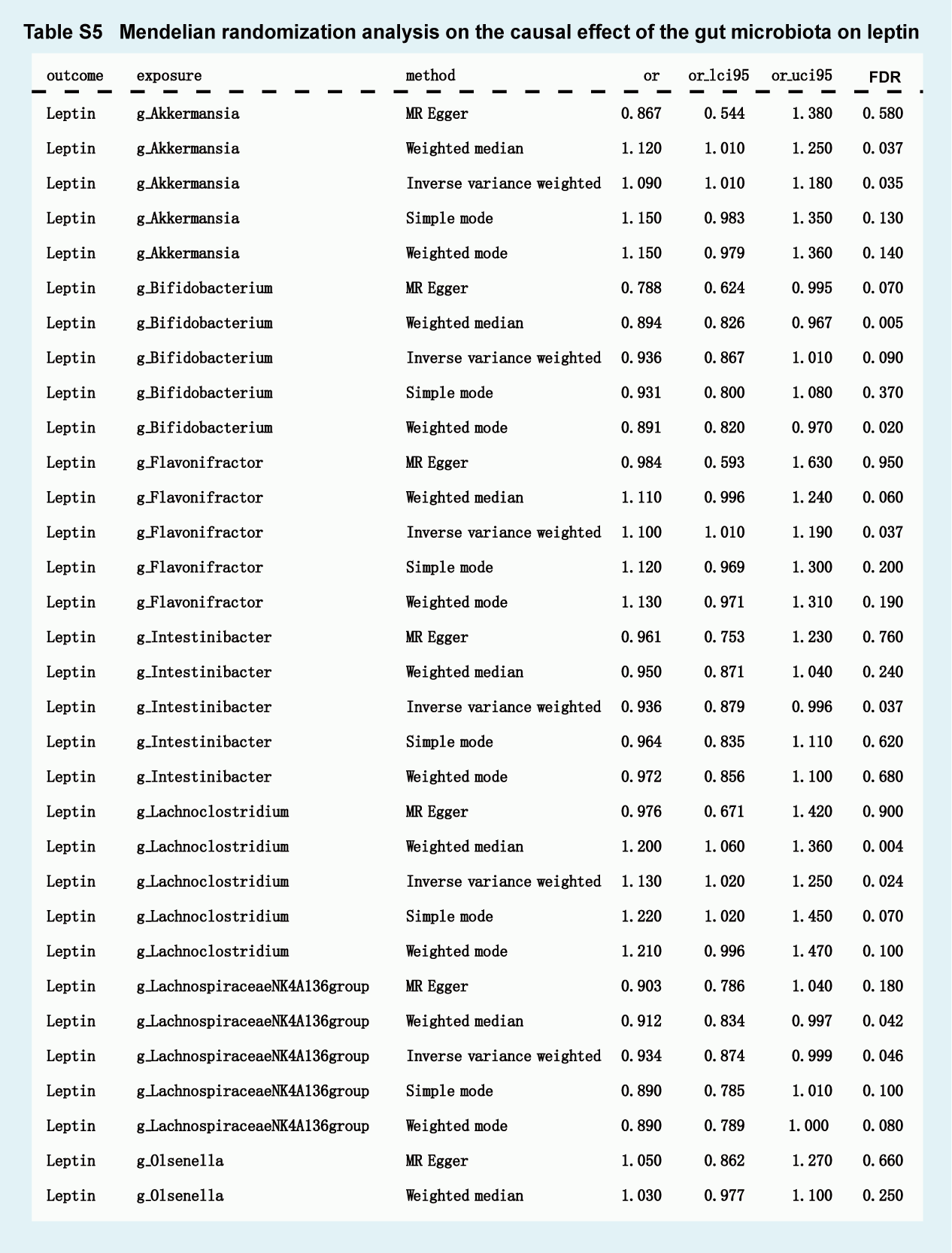


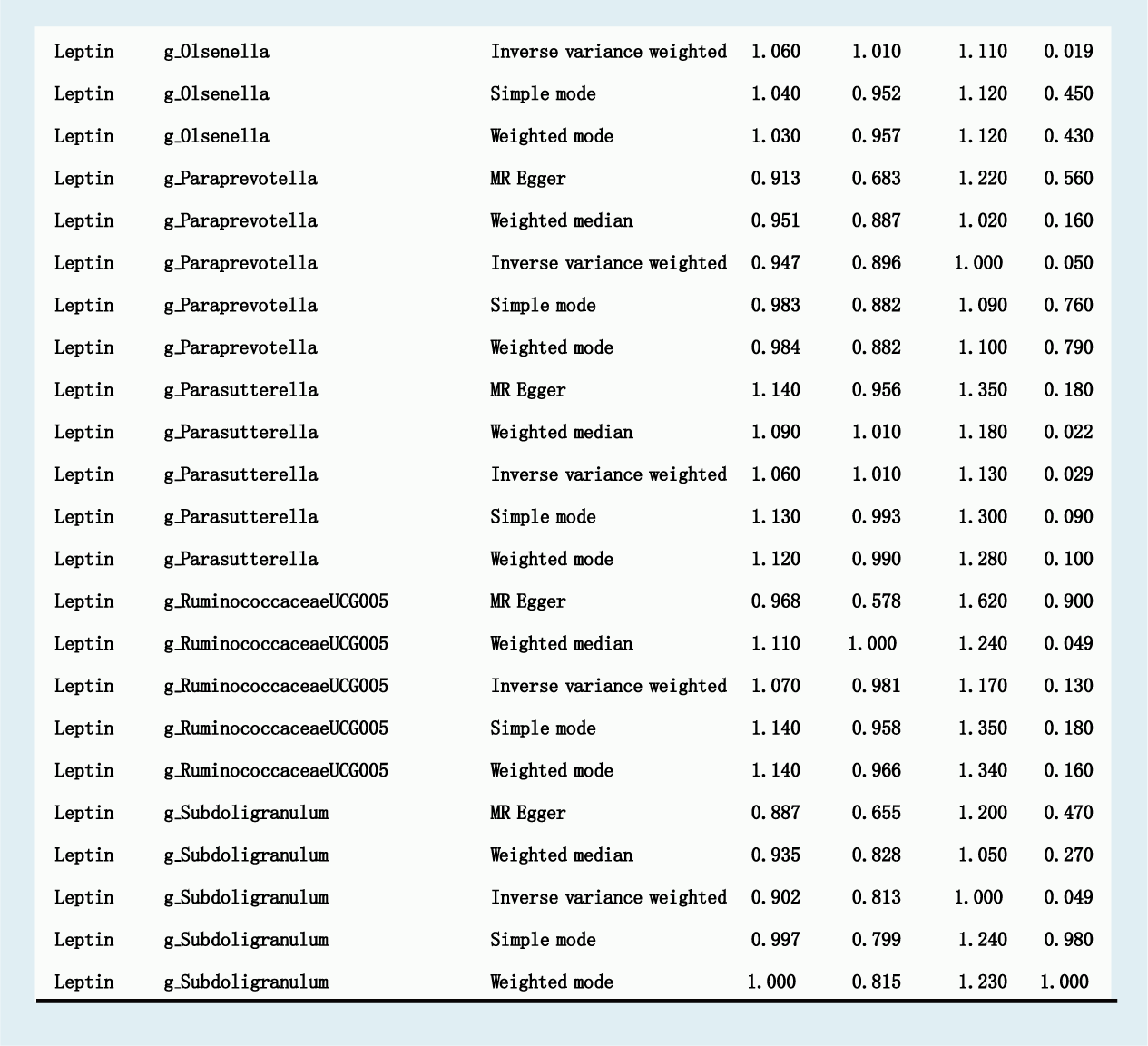


**Table S5. Mendelian Randomization analysis on the causal effect of gut microbiota on leptin**. Inverse variance weighted, Weight median, MR Egger, Simple mode, and Weight mode were used to identify the microbial taxa associated with appetite. Or, or_lci95, or_uci95 and FDR were calculated for the respective method of MR analysis. Or, odds ratio; or_lci95, odds ratio_low confidence interval 95; or_uci95, odds ratio_up confidence interval 95.
