# Supplementary material for: Gut microbiota associated with appetite suppression in high-temperature and high-humidity environments
Source: eBioMedicine. 2023 Dec 16;99:104918. doi: 10.1016/j.ebiom.2023.104918 (PMC10765014; doi:10.1016/j.ebiom.2023.104918)
Supplement: Study Protocol [file mmc3.docx]

**Study Protocol**

Experimental purpose: To explore the biological reasons for the phenomenon of decreased appetite in high humidity and high heat environments, and to demonstrate whether the gut microbiota is a key factor in this phenomenon.

Experimental Plan: This study is mainly divided into two parts. The first part is to obtain the differential microbiota that causes appetite changes in the HTH environment through mouse HTH environment experiment/mouse fecal microbiota transplantation experiment; The second part verifies the reliability of the differential microbiota obtained in the first part through Mendelian randomization analysis and correlation analysis between gut microbiota and appetite hormones.

Animal experiments: We selected Babl/c mice to conduct experiments and observations in a high humidity and high heat environment (humidity 90-95%, temperature 33 ° C ± 1 ° C) for 1, 2, 4, and 8 weeks, and set up corresponding blank control groups. Collect and record the diet and weight of mice on a daily basis, collect corresponding feces, and conduct necessary sampling regularly. After fecal pretreatment, 16S rRNA amplicon sequencing is performed. After blood pretreatment, relevant Elisa tests (mainly detecting appetite hormones GLP-1, PYY, insulin, and hunger) are performed. Other tissues and organs are pre-treated and stored in a refrigerator at -80 ℃.

Fecal microbiota transplantation experiment: We selected Babl/c mice for a 3-week experiment. In the first and second weeks, the mice were infused with 200ml of fecal water daily (feces from the animal experiment DH8W group), and the feeding environment was normal. The fecal microbiota transplantation control group was also set up. The other treatments are consistent with animal experiments.

Validation experiment: In this study, we used Mendelian randomization analysis to focus on the causal relationship between 131 gut microbiota characteristics and appetite changes (particularly manifested as "recent poor appetite or binge drinking"), as well as certain appetite hormones (leptin, PYY, GLP-1, and insulin). Spearman correlation was used to evaluate the correlation between adjacent homones and gut microorganisms.
