# Supplementary material for: Gut microbiota associated with appetite suppression in high-temperature and high-humidity environments
Source: eBioMedicine. 2023 Dec 16;99:104918. doi: 10.1016/j.ebiom.2023.104918 (PMC10765014; doi:10.1016/j.ebiom.2023.104918)
Supplement: Reagent Validation [file mmc4.docx]

**Reagent validation**

| Name | Supplier name | Company | Catalog Number |
| --- | --- | --- | --- |
| Ghrelin | BGsciences Biotechnology Co., Ltd | Elabscience | E-EL-M0551c |
| Insulin | BGsciences Biotechnology Co., Ltd | Elabscience | E-EL-M1382c |
| GLP-1 | BGsciences Biotechnology Co., Ltd | Elabscience | E-EL-M3012 |
| PYY | BGsciences Biotechnology Co., Ltd | Elabscience | E-EL-M2375c |
